# Integrating Digital Technologies and Public Health to Fight Covid-19 Pandemic: Key Technologies, Applications, Challenges and Outlook of Digital Healthcare

**DOI:** 10.3390/ijerph18116053

**Published:** 2021-06-04

**Authors:** Qiang Wang, Min Su, Min Zhang, Rongrong Li

**Affiliations:** School of Economics and Management, China University of Petroleum (East China), Qingdao 266580, China; B19080004@s.upc.edu.cn (M.S.); s20080047@s.upc.edu.cn (M.Z.)

**Keywords:** public health, digital technologies, COVID-19 applications, challenges, China

## Abstract

Integration of digital technologies and public health (or digital healthcare) helps us to fight the Coronavirus Disease 2019 (COVID-19) pandemic, which is the biggest public health crisis humanity has faced since the 1918 Influenza Pandemic. In order to better understand the digital healthcare, this work conducted a systematic and comprehensive review of digital healthcare, with the purpose of helping us combat the COVID-19 pandemic. This paper covers the background information and research overview of digital healthcare, summarizes its applications and challenges in the COVID-19 pandemic, and finally puts forward the prospects of digital healthcare. First, main concepts, key development processes, and common application scenarios of integrating digital technologies and digital healthcare were offered in the part of background information. Second, the bibliometric techniques were used to analyze the research output, geographic distribution, discipline distribution, collaboration network, and hot topics of digital healthcare before and after COVID-19 pandemic. We found that the COVID-19 pandemic has greatly accelerated research on the integration of digital technologies and healthcare. Third, application cases of China, EU and U.S using digital technologies to fight the COVID-19 pandemic were collected and analyzed. Among these digital technologies, big data, artificial intelligence, cloud computing, 5G are most effective weapons to combat the COVID-19 pandemic. Applications cases show that these technologies play an irreplaceable role in controlling the spread of the COVID-19. By comparing the application cases in these three regions, we contend that the key to China’s success in avoiding the second wave of COVID-19 pandemic is to integrate digital technologies and public health on a large scale without hesitation. Fourth, the application challenges of digital technologies in the public health field are summarized. These challenges mainly come from four aspects: data delays, data fragmentation, privacy security, and data security vulnerabilities. Finally, this study provides the future application prospects of digital healthcare. In addition, we also provide policy recommendations for other countries that use digital technology to combat COVID-19.

## 1. Introduction

The global Coronavirus Disease 2019 (COVID-19) pandemic continues to spread with the increasing number of infection cases [1,2]. Studies have shown that the global pandemic is likely to start a second outbreak in autumn and winter [3,4]. The most severely affected country in the world is the United States, where the death toll caused by COVID-19 accounts for about 20% of the total global death toll from the pandemic [5,6]. According to the statistics, its single-day newly confirmed cases have been hovering at a high level, and the pandemic situation has become increasingly severe [7,8].

China was the first country in the world to experience the COVID-19 pandemic, and also the first country that controlled the pandemic in the shortest time [9,10]. During the first wave of COVID-19, China timely adopted strict non-pharmaceutical intervention measures, which effectively contained the spread of the pandemic and reduced the scale of the outbreak [11,12]. Research shows that without non-pharmacological interventions, by February 19, the 50th day of the outbreak, the number of confirmed COVID-19 cases outside Wuhan will reach 744,000 (±156,000). On February 19, 29,839 confirmed cases were reported outside Wuhan, that is to say, the total number of cases actually occurred was reduced by 96% compared with the situation without intervention measures [13]. In the second wave of COVID-19, small-scale pandemics occurred in some areas in China, such as Beijing [14], Dalian city in northeast China, Qingdao city in east China [15], and Kashgar of Xinjiang in west China [16,17]. Through non-pharmaceutical interventions (NPIs), these localized pandemics were quickly brought under control and hardly affected normal economic activities across China [18].

Existing studies equated China’s successful NPIs to control COVID-19 with obvious measures such as “city lockdown” [19,20,21], and even discuss this issue from a political or ideological perspective, but few systematically and comprehensively investigate the technical factors behind it. In order to make up for the gaps in this research, this work will systematically analyze the technical measures supporting China’s NPIs from the perspective of digital technologies such as big data, artificial intelligence, and 5G. This research will not only investigate the reasons for China’s successful control of COVID-19, but also discuss the challenges and future trends of these digital technologies. Our findings can provide references for other countries that are fighting against COVID-19 and can also provide theoretical support for preventing the second wave of global pandemic rebound.

The rest is arranged as follows: Section 2 describes the origin and evolution of digital health; Section 3 is the bibliometric analysis of digital medical research; Section 4 provides evidence that digital technologies are applied to combat COVID-19 in China; Section 5 reveals the limitations of digital technologies in fighting against the pandemic; Section 6 discusses the future development trend of digital technologies in the future and puts forward policy implications.

## 2. Background Information

### 2.1. The Concept of Digital Health

Digital health covers eHealth, mHealth and emerging fields. Mobile health (mHealth) refers to the provision of health services and information through mobile technology, and mHealth belongs to electronic health (eHealth) [22]. The concept of eHealth precedes digital health. E-Health refers to the cost-effective and safe use of information and communication technologies in the health field (such as healthcare services and health monitoring). Although digital health originates from eHealth, it goes far beyond eHealth. Digital health extends the concept of eHealth to more smart and connected devices for digital consumers. A large number of digital technologies such as the Internet of Things and artificial intelligence (AI) are widely used in health care [23]. Telecare, telehealth, telemedicine, mHealth, digital health and eHealth services are collectively referred to as technology enabled care, which integrates medical technology, digital, media and mobile communications.

Sonnier included genomics into the definition of “digital health” for the first time. He believes that digital health is the integration of digital and genomic revolution with health, medical care, life and society [24]. Rowland has further summarized the definition of digital health. He believes that digital health is health and medical care in a digital society. Digital health takes the citizen as the center, collects data in real time from all social activities, and uses complex analysis to obtain knowledge from these data to intervene in the widest possible social and economic activities [25]. In 2019, the World Health Organization (WHO) merged related concepts such as medical informatization, mobile medical, mobile health, and digital medical into “digital health” [26].

In short, digital health refers to using digital health technologies to improve people’s health and provide basic services. Broadly speaking, digital health is a cross-discipline covering the entire medical research field, covering a wide range and application. In a narrow sense, digital health refers to the research and application of digital medical technology, that is, the full use of computer science and digital technologies to carry out new exploration and innovation within the scope of clinical medicine.

### 2.2. The Digital Technologies Behind Digital Health

The development of digital health is inseparable from digital technologies, such as the Internet of Things (IoT), artificial intelligence (AI) and cloud computing. These technologies are used interactively during the application process, rather than simply exist independently. This work mainly lists several key data technologies in digital healthcare.

#### 2.2.1. Internet of Things

Ashton at Massachusetts Institute of Technology (MIT) first proposed the concept of the Internet of Things in 1999 [27]. The IoT refers to the interconnection of all ordinary objects that can perform independent functions through various information sensing devices. The application of the IoT in the medical field has great potential [28]. The IoT can help hospitals realize intelligent management [29], collect and share data such as drugs and medical information. The drug identification system of the IoT can reduce human-induced medication errors and improve the work efficiency of medical staff [30].

The IoT technology can reduce medical costs [31,32]. With the advent of an aging society, the number of patients suffering from chronic diseases is increasing. For patients with chronic diseases, the economic cost and time cost of long-term care work is a major burden for the family. The IoT technology can realize automatic monitoring and tracking of patients, medical staff and medical equipment, and remotely monitor the physical condition of patients [33]. With the help of the Internet, doctors and nurses can monitor the physical condition of patients in real time and manage patients remotely. If the patient’s physical data is abnormal, the alarm system can notify the medical staff to diagnose the patient as soon as possible to ensure the patient’s health [34].

#### 2.2.2. Artificial Intelligence

In 1956, McCarthy first proposed the concept of AI [35]. AI technology can simulate human decision-making and reasoning processes, and supplement and enhance human intelligence through continuous machine learning [36]. AI involves computer science, mathematics, cognitive science, neurophysiology, information theory and other fields, including expert systems, machine learning, natural language processing, automatic planning, image processing and other technologies [37]. AI can automatically learn from massive data to obtain knowledge, and make accurate predictions based on the results of data learning. AI can provide medical staff with auxiliary diagnosis and auxiliary treatment, reducing the workload of medical staff, which improves the work efficiency and medical service level of medical staff [38,39].

AI-based intelligent systems are used in many aspects of the medical field, of which imaging inspection is the most widely used. Many hospitals use AI medical image recognition systems to analyze a large amount of image and diagnostic data to achieve feature extraction of medical images [40]. These image features are used to determine whether there are lesions in the human body, and to further classify and identify the lesions to improve the efficiency and accuracy of diagnosis [41]. AI-based medical robot is a major advancement in modern clinical medicine. The Da Vinci surgical robot system represents the highest level of today’s surgical robots. The system can take pictures of the surgical process through a 3D vision system and complete some operations that cannot be done manually [42].

#### 2.2.3. Blockchain

Blockchain technology is widely used in electronic medical record management, drug and drug supply chain management, health data analysis, etc. [43,44]. Blockchain technology is the basic technology of bitcoin, which is essentially a decentralized database technology. Blockchain has the characteristics of decentralization, openness, anonymity, and non-tamper ability [45]. With the continuous development of electronic health technology, medical health data has increased rapidly, and problems such as difficulty in data sharing between medical-related institutions and easy leakage of data privacy have emerged [46]. Blockchain stores data in the medical system, and adds smart contracts to manage patient data, realizing the safe storage and data sharing of medical data [47,48,49,50].

The decentralization of blockchain makes the rights and obligations of any node equal, and it is impossible to tamper with data through illegal means of controlling the central node. The non-tampering feature of the blockchain improves the security of medical data. The tamper-proof and decentralized functions of the blockchain ensure the security of electronic medical and health records and protect the privacy of patients. The anonymity and openness of the blockchain make the acquisition of medical data more transparent, without the need for real names to gain trust [51]. The smart contract mechanism of the blockchain ensures the privacy and data control rights of users and provides effective access mechanisms and services for users to store and access data in the blockchain [52]. In the blockchain system, only authorized parties have the right to access the patient’s private data, which gives users a high degree of control over personal medical data and guarantees the authenticity and integrity of medical data to the greatest extent. In addition, payment transactions between medical institutions and patients are realized through smart contracts. Smart contracts reduce the occurrence of claim fraud and can establish a trust mechanism without relying on third-party intermediaries.

#### 2.2.4. Cloud Computing

Cloud computing is a disruptive technology, which has developed into a hot technology [53]. Cloud computing is a computing resource delivery model and a type of distributed computing technology. Cloud computing technology integrates various servers, applications, data and other resources, and provides these resources in the form of services through the network, making computing resources virtual. The cloud architecture can be divided into four layers: data center, infrastructure, platform and application [54]. Cloud computing services provide the possibility for users to access and process information anytime and anywhere [55]. And using a lot of cloud resources can reduce costs and improve resource utilization efficiency.

Cloud computing can improve medical services and is conducive to medical care research [56]. The low cost of cloud computing makes various security measures (such as hardware, software, human resources, and management costs) cheaper when implemented on a large scale. Cloud service providers can replicate user data in multiple locations, eliminating errors in manual data collection and reducing the cost of data collection. Cloud computing enables real-time and efficient data sharing [57].

Cloud computing provides convenience for IoT data collection and data processing [58,59]. The combination of cloud computing and the IoT provides many innovative services for the medical field. For example, sensors connected to medical equipment can collect important patient data and transmit the data to the cloud in the medical center for storage and processing [60]. Cloud services can properly manage the information provided by sensors and ensure that medical data such as electronic medical records can be accessed or shared anytime and anywhere [61]. In addition, it makes the process of collecting and delivering data easy to automate, reducing management costs [62].

#### 2.2.5. Big Data

Big data refers to a collection of data whose content cannot be captured, managed, and processed with conventional software tools within a certain period of time. Specifically, big data has five basic characteristics: huge data volume (Volume), diverse data types (Variety), fast processing speed (Velocity), low value density (Value) and high data authenticity (Veracity). Big data technology refers to computer technology that quickly obtains valuable information from various types of data.

Healthcare is considered to be one of the most promising business areas for big data applications [63]. Big data has promoted the transformation of traditional medical services to digital medical services. After entering the era of big data, clinical data is growing at an exponential rate, and the great value of medical data is constantly being reflected [64]. Medical big data needs to be retained for a long time, so the required capacity is larger. The provision of medical information services requires a large amount of online or real-time data analysis and processing, so faster generation speed is required. The use of big data technology provides a good solution to these problems. Big data technology accurately analyzes the data collected from electronic medical records and medical equipment, extracts available data, and forms virtual patients or diagnosis and treatment plans. Big data analysis can propose personalized treatment plans according to different patients [65]. Big data technology can analyze personal health, diagnosis and treatment data, and prevent personal diseases.

Big data technology played a great role in the systematic management of hospitals and effectively improved the hospital’s medical quality. Secondly, big data technology can provide doctors with accurate and scientific information, thereby assisting doctors in diagnosis and treatment, reducing the rate of misdiagnosis. Data mining technology can objectively and scientifically analyze various indicators and values of hospital management activities, so it can assist hospital managers in management decisions. A medical scientific research data center based on big data can greatly improve the scientific research capabilities of hospitals, increase the speed of medical data collection and processing, realize scientific research information sharing, and promote the prosperity of medical scientific research.

#### 2.2.6. 5G Communication Network

5G network is the 5th-generation mobile networks, which is the latest generation of cellular mobile communication technology. 5G has the advantages of high speed, large broadband, and low latency, and its data transmission rate is much higher than that of previous cellular networks. Compared with the current 4G technology, 5G technology will increase its peak rate by several tens of times.

5G technology has been applied in many medical fields [66]. 5G can solve the problems of poor real-time signal propagation, low visual field clarity and remote-control delay in remote surgery under 4G network conditions [67]. The doctor can see the scene of the patient’s operation in real time through high-definition audio and video, grasp the real-time data of the operation, and realize the remote operation of the operation. With the help of the high-speed transmission of the 5G network, the experts can understand the progress of the operation and the patient’s condition in real time during remote collaborative operations, ensure the stability, reliability and safety of the operation, and effectively reduce the risk of the operation. In remote areas, the level of medical care is low. Experts can use 5G technology to conduct remote consultation and remote clinical diagnosis, so as to provide patients with better treatment plans.

### 2.3. Digital Tool of Digital Health

#### 2.3.1. Mobile Devices

As an important tool of digital medical care, mobile devices have penetrated into our lives [68]. Mobile devices are considered to be computer devices that can access various information anytime and anywhere and provide many advantages in the electronic health process. The portability of mobile devices makes it possible to provide medical care services anytime and anywhere without being restricted by geographic location and time and has high practicality. The role of mobile devices in the medical field mainly depends on applications. The rapid development of digital medical care leads to a sharp increase in the number of various health applications [69]. The applications of mobile devices contain sensors, which are mainly used to identify and collect users’ health data. For example, mobile devices can collect the parameters of the user during exercise and detect the user’s sleep and eating status to prevent diseases [70,71,72].

More and more patients and caregivers use mobile devices to share medical information and practical experience online. Users learn health care knowledge and perform self-care management through medical applications in mobile devices [73]. The government and medical institutions push medical knowledge to citizens through apps. During the outbreak of the COVID-19, a large number of mobile applications push the latest news of the pandemic and the latest government measures to ensure that residents can grasp the development of the pandemic in real time. Additionally, these apps also support healthcare professionals to check the health of patients. Many hospitals launched online outpatient appointments, online diagnosis and other services. Patients can make appointments for diagnosis in advance through the application on their mobile devices, which not only saves the time of patients and doctors, but also improves the efficiency of treatment.

#### 2.3.2. Wearable Devices

Wearable devices with biosensors as the core can be directly worn on the body, and can sense, record, analyze, and manage health data with the support of related applications. These wearable sensors can collect physiological signals of the human body and carry out long-term continuous health monitoring [74]. The monitoring data can help users grasp the process of dynamic changes of physiological indicators and provide a more adequate basis for clinical diagnosis. These data can also be used to predict diseases, realize early detection and early diagnosis of diseases, and reduce medical costs for individuals and society [75].

Smart watch is the most common wearable device, which has the functions of monitoring and information processing. The Apple Watch is a typical smart watch with health services. Its functions include sleep detection, heart rate measurement, blood oxygen detection, exercise monitoring, and fall detection. The health information collected and recorded by the smart watch can be shared with third-party medical service institutions with the permission of the user. Users can adjust their living conditions according to the detection data of the smart bracelet to improve the quality of healthy life.

Smart belt is also one of the common wearable medical devices. The smart belt can not only monitor the user’s sitting posture at all times, but also track the user’s activity time and sleep time. Active Protective, a healthcare start-up company, has developed a smart belt that not only detects the fall of the elderly, but also adds a miniature airbag to the belt to ensure that it can cushion the elderly when they fall and avoid hip fractures [76].

Wearable blood glucose monitors can perform regular checks on patients to help diabetic patients monitor and control blood glucose levels. This wearable device can continuously monitor the user’s blood glucose concentration level, formulate reasonable and effective diabetes management strategies based on the monitoring data, and prevent long-term complications and diseases [77].

Smart pill for drug delivery is one of the important digital medical applications. Smart pill refers to oral medications containing tiny ingestible sensors, which can automatically release the ingredients of the medication in the patient’s body and generate feedback signals. Smart pills are applied to monitor patient compliance with prescription drugs. Helius pill is a smart pill that contains very small sensors. The pill is activated after the patient takes it, and the feedback signal is received by a device attached to the surface of the patient’s skin. The Helius system uses wireless transmission technology to transmit medication information to the corresponding receiver, thereby tracking the time and dose of medication and monitoring the user’s heart rate and body temperature [78].

#### 2.3.3. Surveillance Cameras

Digital technologies have increased the potential of remote monitoring, and surveillance cameras play an extremely important role in telemedicine. Promoting the construction of an intelligent monitoring system for medical services can alleviate the problem of uneven distribution of medical resources. Remote monitoring helps to monitor the health of patients outside of the routine clinical environment, which improves the work efficiency and quality of doctors, and saves patients a lot of treatment time and costs. Using surveillance camera equipment, medical institutions and medical personnel can digitally process and reconstruct medical images to achieve remote image acquisition, storage, transmission and analysis for remote diagnosis and treatment of diseases [79].

In terms of nursing care, monitoring equipment can enhance nursing work. Continuous and accurate remote monitoring is conducive to improving the quality of care and allowing patients to enjoy greater freedom outside the hospital [80]. The monitoring equipment has the ability to analyzes and processes the patient’s respiratory rate, pulse, blood pressure and other physiological information, and then transmits the analysis results to clinicians and other systems. Medical staff diagnose diseases and give treatment plans based on the physiological parameters obtained, which further improves the level of medical services [81].

## 3. Bibliometric Analysis of Digital Medical Research

### 3.1. Methods and Data

The data in this paper comes from the Scopus database, which is a comprehensive literature abstract and citation database. Scopus provides scientific research results in the fields of science, technology, medicine, social sciences, art and humanities. To sort out the development and current situation of digital medical care, the paper carried out searches according to the search formula given in Table 1 around the two themes of “digital medical” and “digital medical during COVID-19”.

**Figure 1:**
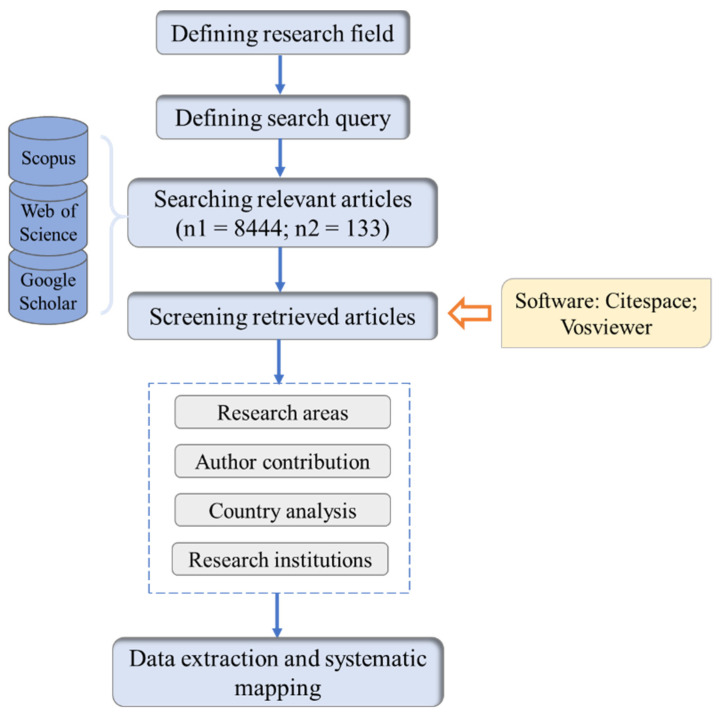
Steps of visual analysis through Cite Space and VOS Viewer.

The specific analysis process is shown in Figure 1. VOS Viewer and Cite Space are applied to create visual maps. CiteSpace is an information visualization software developed by Chen using Java language. It presents the structure, regularity and distribution of scientific knowledge through visualization, and then obtains a map of scientific knowledge [82]. VOS viewer is a software tool used to build a visual bibliometric network. These visual networks include journals, researchers or personal publications. VOS viewer can also build a relationship network based on citation, bibliographic coupling, co-citation or co-author relationship. VOS viewer also provides text mining functions to construct and visualize co-occurrence networks of important terms extracted from scientific literature [83].

### 3.2. Bibliometric Analysis

#### 3.2.1. Descriptive Analysis

**Figure 2:**
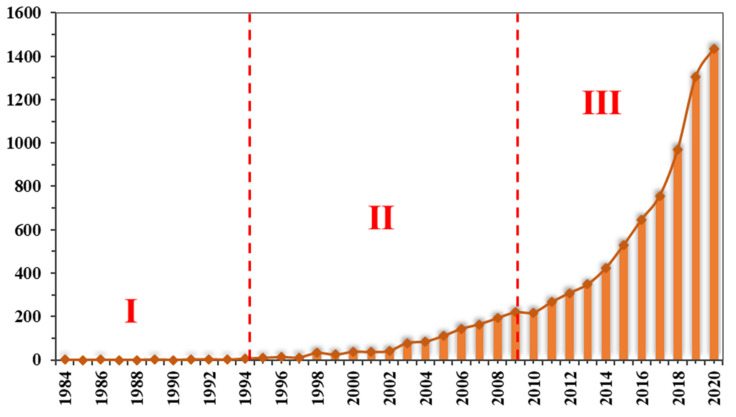
The number of papers published annually (until November 2020).

As of 18 November 2020, the Scopus database contains 8444 documents related to digital healthcare. Figure 2 shows a graph of digital medical research over time. Since the statistics for 2021 are not yet complete, they are not included in the analysis. From Figure 2, the first publication on digital medicine was published in 1984. The paper titled “40th International Conference on Information Systems” aimed at the safety and privacy of patients and proposed the use of blockchain technology to build a digital health network [84]. From 1984 to 1995, the number of publications increased slowly, and the number of publications did not exceed 10 per year. During the period 1996–2010, the number of publications gradually increased, but the growth trend was relatively slow. In 2010, the number of papers related to digital medical treatment exceeded 200, which means that digital medical research has entered a new stage.

From 2011 to 2020, the number of publications has grown rapidly, with an average annual growth rate maintained at around 20%. This shows that digital healthcare has received more widespread attention from the academic community. The publications in 2020 has exceeded 1400, which may be related to the COVID-19 pandemic. After the WHO announced that COVID-19 has become a Public Health Emergency of International Concern (PHEIC) on 30 January 2020, governments and research institutions around the world have carried out relevant research [85]. A report from China shows that the widespread use of digital communication devices (such as mobile communications and walkie-talkies) shortened the time that medical staff have close contact with patients, reduced the risk of nosocomial infection, and also reduced the use of medical protective equipment [86]

**Figure 3:**
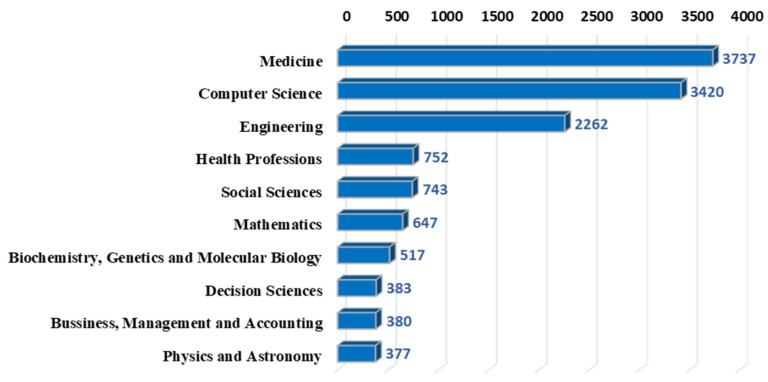
Top 10 subject categories in healthcare.

Figure 3 shows the top 10 subject categories involved in digital healthcare. Medicine, computer science, and engineering are the three fields with the largest number of publications. On the second gradient are health professions, social sciences, and mathematics. At the current stage, the medical field and the computer field are the areas where digital healthcare publications are mainly concentrated, which may be related to the processing objects and operating modes. In terms of processing objects, digital healthcare has a wide range of clinical applications, such as breast cancer screening [87], cardiopulmonary resuscitation [88], and otitis media treatment. For operating modes, from early telephone video to blockchain and big data [89,90,91], the development of information systems have affected the practice of digital healthcare [92]. Furthermore, social sciences, biochemistry, genetics and molecular biology also have a lot of publications.

#### 3.2.2. Cited Authors Analysis

Co-citation analysis means that two articles appear together in the reference list of the third article, and the two documents form a co-citation relationship [93]. The author’s co-citation analysis is derived from the paper’s co-citation analysis. In the author’s co-citation network, concentric circles represent the separation of years of publications, and the size of the circles indicates the author’s central position in the collaborative network. Purple indicates older papers, and orange indicates relatively new publications. The author’s co-citation network shows leaders in the field. In this section, the author’s co-citation is analyzed through Cite Space. The time slice is selected as 1 year, and the cutting method is Pathfinder and Pruning the merged network. Intermediary centrality was proposed by American scholar Freeman, and it is an important reference indicator for social network analysis. Intermediary centrality represents the degree of control of a certain network node on surrounding resources in the research network, and the importance of this point in the network is evaluated by studying the frequency of the node as the shortest path intermediary between the other two nodes in the network.

**Figure 4:**
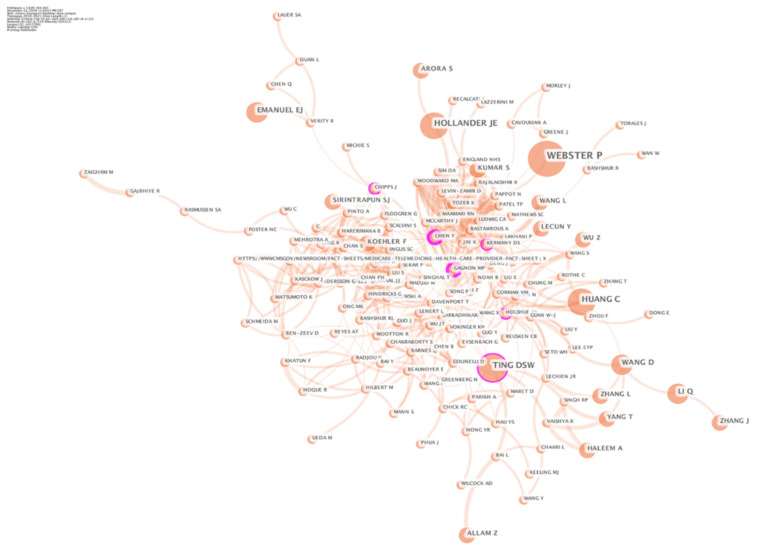
The cooperation network of cited author.

As shown in Figure 4, Gagnon Marie Pierre from Canada, Holshue Michelle L and Kermany Daniel S from the United States, Daniel Shu Wei Ting from Singapore, and Chipps Jennifer from South Africa are co-cited authors with high centrality in this field. The similarity of their research is that they are not limited to the treatment of a certain disease but are committed to the construction of the medical care system, laying the foundation for subsequent research. Among them, Holshue Michelle L’s re-search during the COVID-19 pandemic is worthy of attention. The focus of the re-search is not on disease treatment, but on disease risk management and pandemic prevention and control.

#### 3.2.3. Country Analysis

**Figure 5:**
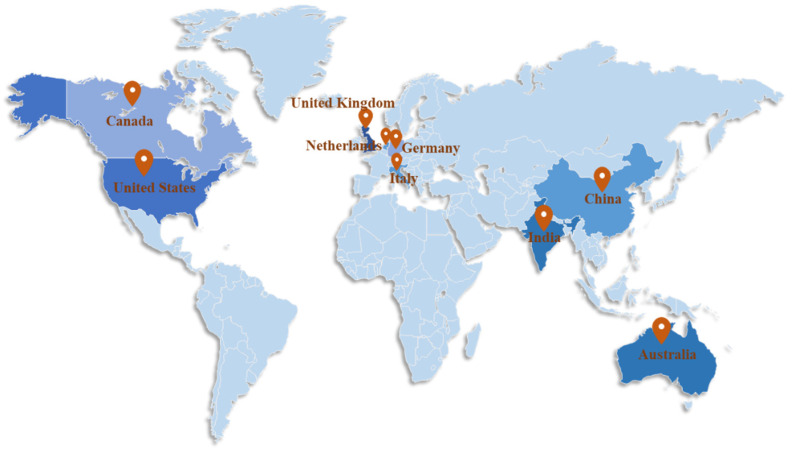
Global geographic distribution of digital healthcare publications.

The regional distribution of the literature is determined according to the author’s institutional address stated in the title of the publication. Figure 5 displays the global geographic distribution of digital healthcare related publications, where color depth rep-resents the number of national publications. In terms of the total number of publications, the United States has the most publications. As of 2019, there are more than 3000 US private companies engaged in digital healthcare, and the market size is around US $10–200 billion [94]. These companies include industry regulatory interaction, payment management, hospital management, patient medical records, medical team collaboration, doctor-patient interaction, clinical technical support, biotechnology and other aspects, which to some extent represent the United States’ leadership in this field.

It is followed by United Kingdom, India, Australia, Germany, China, Italy, Canada, and Netherlands. Comparatively speaking, developed countries have more advantages in digital healthcare research. As representatives of developing countries, India and China rank among the top in the world in the number of publications. India has formulated a framework for the development of the national digital healthcare, the “National Digital Medical Development Blueprint” [95]. In China, the “13th Five-Year” National Science and Technology Innovation Plan proposes to focus on the development of emerging service industries such as digital health and digital life [96]. The relevant policies implemented by developing countries to promote the development of digital healthcare played a positive role in increasing the number of publications.

**Figure 6:**
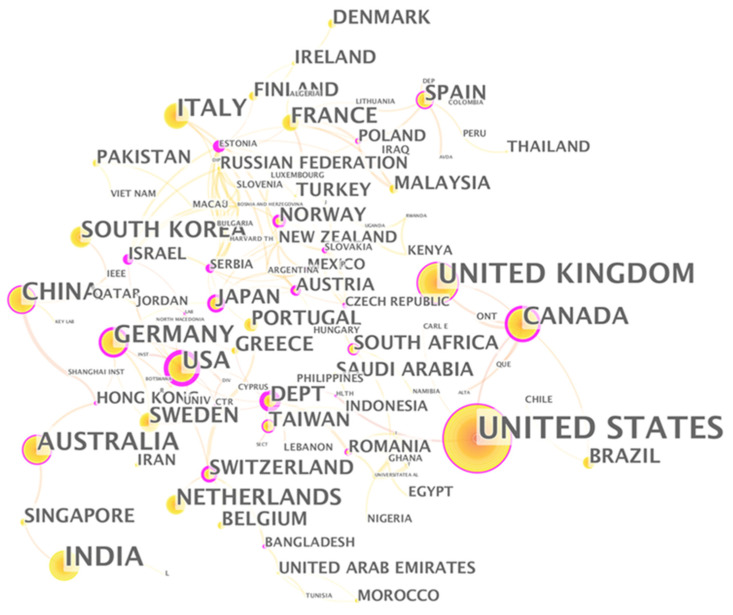
The cooperation network of countries.

Figure 6 is the national cooperation network. Each node in the figure represents the number of papers published in the country where the first author belongs, and the thickness of the line between the nodes represents the strength of cooperation between the two countries. From Figure 6, many countries have formed a complex and close cooperation networks in the field of digital healthcare. The United States, the United Kingdom, Germany, China, and Japan published a large number of papers, and established complex cooperation networks with other countries.

Estonia is one of the countries with high centrality. Estonia has an area of only 45,000 square kilometers and a population of 1.319 million. Compared with the United States, Britain, Germany, China and Japan, Estonia does not have an advantage in terms of resources. But Estonia has been using the latest information technology to promote the process of digital society. With the full promotion of the government, Estonia is one of the most digitally advanced countries in the European Union and has established an efficient, safe and transparent social ecosystem. Encouraging health care providers to use new digital technologies is the key to the success of Estonian e-health policy [97]. In 2008, Estonia established a nationwide electronic medical system to improve the medical quality and efficiency of the public medical system [98]. In 2010, as a supplement to the electronic medical system, Estonia created a centralized paper-less prescription system for the issuance and processing of medical prescriptions. Currently, 99% of medical prescriptions in Estonia are processed online [99].

#### 3.2.4. Funding Agency Analysis

In order to gain insight into the funding agencies in the digital healthcare field, we compiled information on the top ten funding agencies in the Scopus database (Table 2). Funding agencies from the United States, China, the United Kingdom, and European countries pay more attention to digital healthcare research. Public institutions mainly refer to groups and institutions funded by the government, including public universities and government agencies. The table shows that the public sector dominates the top ten funding agencies, which shows that the government has given great support to re-search in response to public health emergencies.

#### 3.2.5. Source Journal Analysis

**Figure 7:**
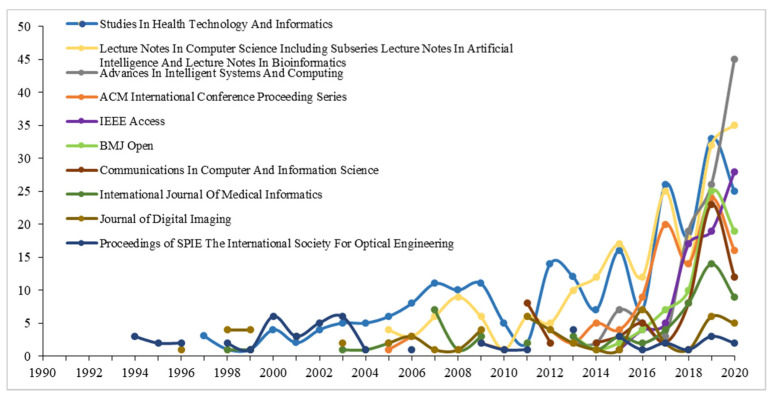
Publications about digital healthcare of top 10 source journals 1990–2020.

Statistics show that scholars published research on digital healthcare in 150 different international journals. Table 3 lists the top ten journals by number of publications. Among the Top10 journals, journals in the computer field dominates, including “Lecture Notes in Computer Science”, “Advances in Intelligent Systems and Compu-ting”, “ACM International Conference Proceeding Series”, “Communications in Computer and Information Science”. Secondly, many publications have been published in medical journals, such as Studies in Health Technology and Informatics and BMJ Open. Figure 7 illustrates the change in the number of annual publications of the top ten journals. Compared with journals in the computer field, the peak of the number of journal publications in the medical field is relatively low, and early publications are mostly published in the computer field.

#### 3.2.6. Keyword Co-Occurrence Analysis

**Figure 8:**
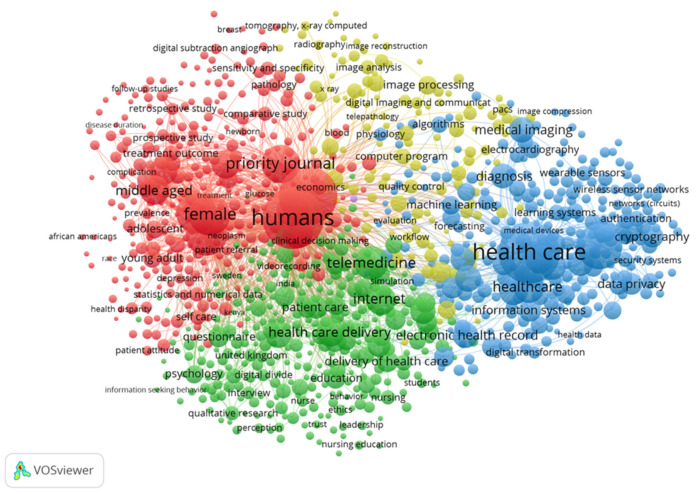
The cluster view of the keyword network on digital healthcare.

Keywords as a refined expression of research topics in academic papers, their relevance can reveal the distribution and evolution of research topics to a certain extent. The key words of the paper can intuitively reflect the changes in hot areas, analytical perspectives, and research methods in different time series, and reveal the internal connections of knowledge in the subject area. VOS viewer is employed to visualize the keyword map in the digital medical field. In Figure 8, similar categories are distributed according to the same color, so the digital medical field can be divided into four main categories:

(1) For the red clusters with humans, female, adolescent, and middle aged as the main keywords, the keyword nodes all have high total link strength. The publications in this part mainly focus on the target groups and research objects of digital healthcare. The age and gender of digital healthcare research objects are relatively wide, and they are not limited to the research of a particular group. Note that “female” has a higher total link strength. Rock Health pointed out in the investment and financing report released in 2019 that the public market for digital medical enterprise IPOs consists of two hot investment areas-behavioral health and women’s health [100]. Women are considered to be the largest group of buyers and decision makers in healthcare.
(2) A green cluster with telemedicine, internet, questionnaire, and digital divide as the main keywords. This group of the keywords mainly focuses on digital medical treatment methods and analysis tools. Blockchain, AI and genome editing are key technologies used in digital medicine. One of the main advantages of blockchain in the field of digital healthcare is that the technology can provide a higher degree of security, privacy, and confidentiality of healthcare data [101]. AI can automatically solve labor-intensive tasks, read radiological images, and analyze patients’ symptoms and vital signs [102].
(3) The blue clusters with data privacy, cryptography, information systems, health record, and medical imaging as the main keywords mainly describe the data technology of digital medical treatment. The emergence of big data provides a cost-effective prospect for the improvement of health care [103]. Many scholars have proposed new digital technologies, such as convolutional neural network (CNN) [104], lot in healthcare [105], electronic medical records (EMR) [106], etc. At the same time, the application of digital technology in the medical field is also facing challenges. On the one hand, the unbalanced development of infrastructure and human capital between developing and developed countries has created a new dimension of the digital divide. These differences hinder developing countries’ attempts to use digital healthcare to provide medical care [107]. On the other hand, the proliferation of big data also brings security and privacy issues [108].
(4) Yellow clusters with image processing, image analysis, and image reconstruction as the main keywords. These keywords are focused on the analysis of medical images. Digital medicine can change the imaging informatics known in the medical world today, thereby providing more accurate, timely and effective treatment plans [109]. As of 2020, the artificial intelligence medical imaging market is the second largest market segment in artificial intelligence medical applications, second only to drug research and development, accounting for 35%.

#### 3.2.7. Keyword Evolution Analysis

Figure 4 displays the evolution of research trends in the digital medical field. Digital medical research has undergone changes in the following four aspects: target groups, business models, technical means and application areas:

First, the target group of digital medical research has shifted from providers to consumers. The initial stage of digital medical care was provider-centric, mainly used to support clinical decision-making, focusing on the integrated interoperability of medical imaging, treatment, and laboratory diagnosis [110]. With the development of digital healthcare, more and more personal health records (PHR) are recognized as a means to support consumer empowerment and value-driven healthcare. People gradually shifted their focus from provider-centric to consumer-centric [111]. Direct-to-consumer digital healthcare shifted from ambiguous purposes and clinical indicators to specific intentions with clear clinical indicators [112].

Second, the business model of digital healthcare has completed the evolution from B2B to C2C to TDHS. In the development of digital medical care, the electronic medical business model can take many forms, each of which has a different purpose, and has its own revenue and cost structure [113]. The most popular business models in the early days were portals, connectivity, business-to-business (B2B). The second stage consumer-to-consumer (C2C) is the main business model. In the third stage, the total digital health system (TDHS) has become a new business model, which can improve the benefits within and between companies by sharing medical information among various medical service providers.

Third, the technical means of digital medical treatment are transferred from computers to intelligent data. The ever-increasing expectations for effective treatment and quality of life are putting increasing pressure on healthcare. This requires science and technology to continuously provide new and more advanced solutions. Intelligent data and data analysis and cognitive computing are promising technologies with huge added value in the medical field [114]. The emergence of technological means such as the Internet of Things, big data, artificial intelligence and cloud computing solved the inefficiency of clinical operations, public health, and drug development [115].

Fourth, the application field of digital medicine has evolved from single disease treatment to providing diversified services. In the early days of digital medical development, telemedicine and remote monitoring greatly eased the pressure on the medical system in effective triage and care, while alleviating the suffering of patients [116]. Subsequently, the digital insurance market and medical e-commerce have gradually emerged [117,118].

**Figure 9:**
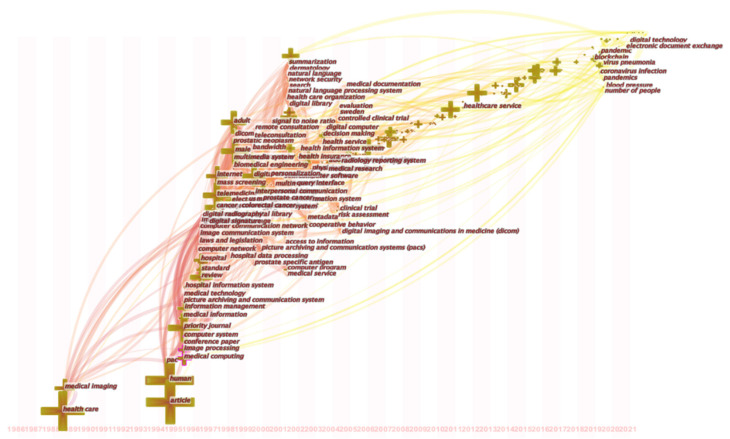
Keyword evolution map on digital healthcare.

Table 4 and Figure 9 lists the top nine health service stocks in the U.S. in 2020. The main businesses of these medical institutions include traditional telemedicine, chronic disease management, equipment leasing and waste disposal. Moreover, third-party services in hospitals also account for a larger proportion.

### 3.3. Digital Healthcare Research during COVID-19

The COVID-19 pandemic is spreading around the world at an unprecedented speed and has attracted widespread attention from scientists from all over the world. The number of publications in the field of digital medicine has increased sharply at this stage, proving the possible impact of public health emergencies on the field of digital medicine. We further analyze the differences in research models and keyword maps in different countries in the context of the COVID-19 pandemic.

#### 3.3.1. Country Analysis

**Figure 10:**
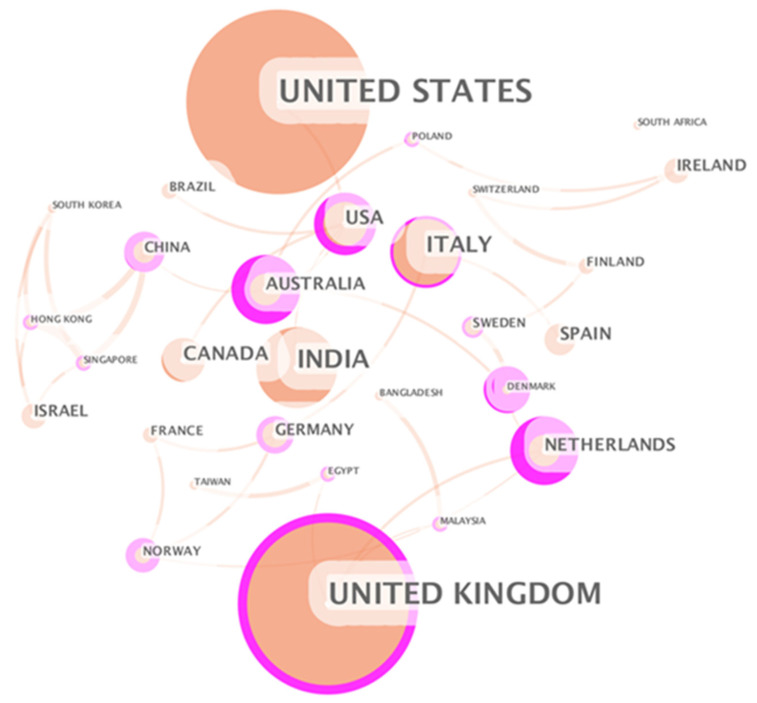
The cooperation network of countries.

The country cooperation map (Figure 10) is generated based on the keywords in Table 1. Comparing Figure 6 and Figure 10, what they have in common is that during the epidemic, major countries such as the United States, the United Kingdom, India, and China played a leading role in digital medical research. The difference is that the role played by research institutes in the digital medicine field in developing countries has weakened during the pandemic, and the most influential countries are mainly developed countries.

The Netherlands shows a more important position in the field of digital healthcare during the pandemic. Taking the Netherlands as an example, we analyze the application of digital healthcare during the COVID-19 pandemic. First of all, the Netherlands is one of the leading countries in Europe in terms of digital healthcare and data management. 87% of patient data nationwide are stored digitally. In February 2020, the Dutch government announced a package of more than 400 million euros to promote cost-effective, connected digital healthcare. The COVID-19 pandemic has further accelerated the innovation and application of digital healthcare in the Netherlands. The planned projects have been accelerated, and a large number of new digital initiatives from the entire medical industry have begun to bear fruit.

#### 3.3.2. Keyword Analysis

**Figure 11:**
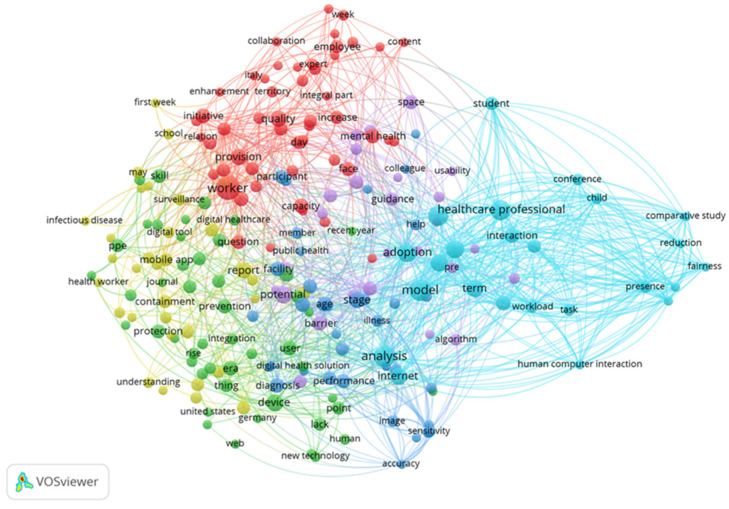
The cluster view of the keyword network on digital healthcare in COVID-19 pandemic.

From Figure 11, the keyword clustering of digital medical publications includes six aspects: (1) Cyan cluster with healthcare professional and model as the main keywords; (2) Red cluster with worker and quality as the main keywords; (3) Potential and mental health as the main keywords purple cluster; (4) blue cluster with stage and facility as the main keywords; (5) green cluster with prevention as the main keyword; (6) yellow cluster with mobile app as the main keyword. In comparison, red clusters and cyan clusters have obvious aggregation, while purple, yellow, green, and blue clusters are staggered in different clusters.

In order to explore the differences between the hot spots of digital medical research before and after the COVID-19 pandemic, this article screened the keywords with higher total link strength in the cluster, and finally obtained six research categories.

(1) Disease risk management and prediction

(a) Pandemic prevention and control. With the rapid development of the covid-19 pandemic, countries all over the world are facing great pressure on the prevention and control of the epidemic situation. It is urgent to improve the efficiency of the epidemic prevention and control. The improvement of efficiency is the advantage of digital healthcare. (b) Tracking. During the COVID-19, residents rely more on mobile communication devices such as smartphones to communicate with the outside world. Therefore, using smartphones and related applications as the starting point of personal information big data will help to identify “potential sources of infection” and effectively monitor the flow and distribution of the population. (c) Remote monitoring. Remote monitoring of patients with COVID-19 can be carried out in a variety of ways [119]. Remote monitoring can optimize the care of COVID-19 patients by detecting clinical deterioration at an early stage. Remote monitoring provides the best care for patients suspected of COVID-19, while avoiding unnecessary hospitalization and reducing hospital stays. It can effectively use scarce medical resources and reduce the risk of further spread of the virus.

(2) Hospital management

The COVID-19 pandemic exposed problems in the manufacturing and supply chain of personal protective equipment in some countries. Cloud computing can improve the computing and storage capabilities of the hospital’s core system. The diversion function of the hospital can be executed digitally, thereby optimizing the matching of human resources, customizing clinical resources, and minimizing the congestion of medical treatment during peak congestion [120]. Medical robots can take care of multiple patients in the quarantine area and take on tasks such as delivering drugs.

(3) Adjuvant treatment

Telemedicine mainly includes mobile health and auxiliary treatment. Mobile health is about medical care, data sharing and improving patient experience through secure mobile applications. Medical staff collect data (such as blood pressure, body temperature, etc.) from patients for analysis by medical practitioners located in different geographical locations. Auxiliary treatment refers to the services of providing medical consultation, self-diagnosis, and diagnosis to users through medical robots [121]. During the COVID-19 pandemic, compared with face-to-face contact and work, the use of digital Technologies to solve the needs of patients for medical and healthcare services has ensured the safety of medical staff [122].

(4) Health Management

Health management includes nutrition, physical health management, and spiritual management. The COVID-19 pandemic may cause emotional loss, loss of self-worth, loss of motivation and other mental health problems. Take students as an example. During the pandemic, the demand for online teaching and the uncertainty of the pandemic aggravated students’ mental health problems. Integrating the digital ecosystem of the school and the medical system through digital Technologies enables schools to directly intervene students and parents through specific evidence-based interventions [123]. In addition, during the blockade, online media and other services reduced some harmful effects, such as loneliness, mental stress and other problems [124].

(5) Complementary medical research

(a) Knowledge training. Academic institutions provide knowledge training for the health system and front-line medical staff, with the aim of cultivating qualified staff for healthcare services [125]. (b) Drug development. The spatial structure of the new coronavirus gene sequence is complex, and the genome is long, which brings difficulties to the development and marketing of new vaccines and new drugs [126,127]. Intelligent digital medicine can be used for drug research [128], providing efficient tools for new drug synthesis route design, drug effectiveness and safety prediction, drug molecular design, and research on new combination therapies. The use of digital tools can not only reduce the cost of drug development, but also shorten the time for new drug development [129].

(6) Information data management

(a) Blockchain. During the COVID-19, data such as personal physical sign information and medical consultation information have grown rapidly. The data has many dimensions and lacks integration, and the quality is uneven. Blockchain has great potential in ensuring the openness and transparency of pandemic information and the tracking and traceability of pandemic materials. Smart contracts based on blockchain technology can be used to automate the audit process and improve drug supply chain management [130]. The use of blockchain technology can manage electronic medical record prescriptions (HER) and reduce clinical deviation [131,132,133]. (b) 5G applications. The high-speed connection and seamless user body of 5G communication enable data sharing in a more complex information ecosystem [134] to create a safer post-COVID-19 world [135]. As shown in the Table 2, digital medical services have strict requirements on technology and require the use of complex basic technologies to achieve normal functions. Therefore, 5G technology is widely used to promote the fight against COVID-19.

## 4. Applications and Impacts of Digital Technology in Fighting COVID-19

The COVID-19 pandemic is considered as the biggest global public health crisis after the 1918 pandemic [136,137]. There is no effective vaccines or treatments for this disease. Considering the high spread of the pandemic, many regions adopted NPIs to curb the spread of the SARS-CoV-2 with varying degrees of success [138,139]. These NPIs mainly refer to digital health plans based on digital technology, and their wide applications in the medical field provides technical support for the government to combat the COVID-19 pandemic [140,141,142]. Digital technology played an irreplaceable role in reducing deaths caused by COVID-19 [143,144,145].

### 4.1. Digital Technology in Epidemic Surveillance

#### 4.1.1. Epidemic Screening and Case Identification

Identifying infected cases quickly and accurately is one of the effective means to curb the spread of the COVID-19 [146]. Experts from the World Health Organization pointed out that the development of the COVID-19 pandemic has increased rapidly, but the deceleration has been much slower [147]. Therefore, timely detection and isolation of COVID-19 carriers is an important way to prevent the further spread of the epidemic [148,149]. Early detection of COVID-19 patients can reduce the spread of the virus and the scale of infection, effectively reducing the infection rate and mortality rate [150,151]. Studies have shown that NPIs such as epidemic surveillance, epidemiological investigations, and tracking the trajectory of close contacts can help detect and report suspicious and infected cases as soon as possible [152,153]. Big data analysis and artificial intelligence provide technical support for epidemic screening such as personal information collection, trajectory query, and human temperature measurement [154,155,156,157].

In the early stage of the epidemic, information reporting was the only source of big data, so it required grassroots staff to manually collect relevant data, which was a huge workload and was easy to miss. In China, government departments and Internet companies have jointly developed a variety of applications to monitor the health of the public and personal behavior in real time, with the goal of accurately tracking and managing the flow of people [158]. Residents report personal information through digital tools, including personal identification information, daily body temperature, personal whereabouts, traffic data, etc. [159,160]. These digital tools are all based on real data and use big data technology and complex algorithms to intelligently determine whether the user is a confirmed case or a potential source of infection [161,162].

The key to curbing the spread of the COVID-19 pandemic is to quickly identify virus carriers in the population, isolate and treat them, and lock their close contacts for isolation and observation. Therefore, the health data collection and activity range monitoring of the population in public areas (including body temperature records, travel trajectories, close contact records, etc.) are very important. The current 4G network bandwidth cannot meet the real-time transmission and storage requirements of massive HD image data and dynamic trajectory data [163]. During the COVID-19 pandemic in China, 5G technology combined with artificial intelligence, biometrics and thermal imaging technology achieved rapid temperature measurement of large-scale mobile populations in multiple railway stations across the country and can quickly identify the wearing of masks. Intelligent temperature measurement of the population not only avoids cross-infection, but also greatly improves the detection efficiency [164,165].

Fever is one of the main symptoms after infection [166,167]. Temperature monitoring in densely populated areas (such as train stations and subway stations) has become an important measure to detect potential infection cases and block the rapid spread of the epidemic. However, the traditional body temperature detection method still has certain limitations. In a traffic scene with a large flow of people, it needs a lot of manpower to use the traditional forehead temperature measurement gun. Moreover, large-scale body temperature detection is likely to cause personnel to stay, and there is a risk of personnel gathering and cross-infection [168]. Therefore, infrared human body thermal imaging temperature measurement systems are used for group temperature measurement in public places such as airports, stations, hotels, and other places with a large flow of people [169,170].

Artificial intelligence technologies such as face recognition can detect suspected cases in time [171]. In traditional access control, turnstiles and other facility scenarios, residents usually enter the community by punching cards and fingerprints, which poses a security risk of infection through indirect contact. In order to eliminate these hidden dangers, during the epidemic prevention and control period, face recognition access control is widely used in the entrance recognition of smart communities. The smart community installed a smart camera with face recognition function at the entrance, which is not only used to open and close the door, but also involves the confirmation of the individual trajectory of the residents. These smart cameras can accurately identify households through AI technology and the big data platform of image collection, which greatly improves the efficiency of personnel passage. This measure solves the problem of inconsistency in the past, brought great convenience to residents, and improved the intensity and efficiency of community epidemic control [172].

#### 4.1.2. Contact Tracking

**Figure 12:**
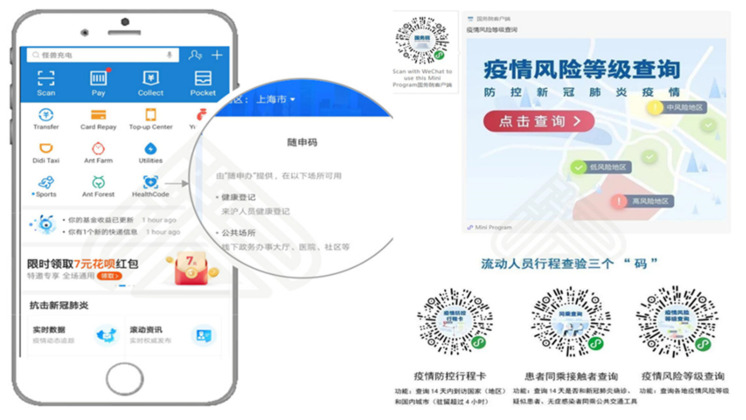
A screenshot of fellow passenger query tool in China [180,181].

The new coronavirus is extremely contagious. Studies have shown that the incubation period of the new coronavirus ranges from 1 to 14 days, and the average incubation period is about 5 days [173,174,175]. 97.5% of infected persons will have clinical symptoms within 11.5 days. Confirmed cases of new coronary pneumonia are infectious for 14 days before the onset of disease [176,177]. As a result, people who have shared public transportation and close contacts of confirmed cases in the past 14 days may be infected and become potential sources of infection. To this end, the National Health Commission and China Electronics Technology Group jointly established a big data research team for epidemic prevention and control, and jointly developed a big data platform for risk population perception-the “close contact measuring instrument” (Figure 12) [178]. This client uses big data fusion analysis to quickly and accurately identify “close contacts” from massive amounts of data, which has three major characteristics: authoritative data, credible models, and accurate queries [179].

In addition, the person-to-person relationship analysis of confirmed cases is also one of the important contents of close contact tracking. Human society is composed of a complex network of social relations. The use of data mining methods such as association analysis, knowledge graphs, and complex network analysis can quickly and comprehensively analyze the interpersonal network of confirmed cases, helping to find transmission paths and hidden transmission nodes [182,183]. Accurate virus traceability helps to accurately locate the source of infection and effectively cut off the route of transmission [184]. Regional isolation is the key to curbing the rapid spread of the COVID-19 [185]. Effective contact tracing helps to detect suspected cases in time to prevent further spread of the epidemic [186].

During the COVID-19, China adopted strict lockdown measures and implemented home isolation measures for residents based on the itinerary of the past 14 days. How to judge whether residents need to be isolated and to inquire about their personal deeds? Big data query and digital tracking provide a good solution to this problem. The epidemic management department inquiries about personal whereabouts in the last 14 days based on the GPS positioning function on the user’s mobile phone. Furthermore, management personnel consider people who have been to the outbreak site in the past 14 days and have symptoms such as fever and fatigue as key risk groups, and professional organizations will conduct sampling and testing on them. The big data platform provides technical support for the early detection and reporting of suspected COVID-19 cases and standardizing the management of close contacts [187,188,189]. Besides, the 12306-ticketing platform uses the big data advantages of real-name ticket sales to timely cooperate with local governments and prevention and control agencies at all levels to provide information on close contacts of confirmed patients on vehicles. If there are confirmed or suspected passengers on the train, passenger-related information, including train numbers, carriages, etc., will be retrieved, and then provided to the relevant epidemic prevention department for follow-up processing.

Cell phone data can play a key role in tracking the movement of people to help determine where the disease may spread. For example, location statistics can help analyze the spread of diseases and help allocate resources based on population distribution. Apple and Google jointly launched an application called “contact tracing” for public health agencies to track close contacts of COVID-19. It can be installed on smartphones (including Apple’s iOS and Android operating systems), using short-range Bluetooth signals to collect data from other mobile phones that have been in close contact with the user, and send it out when people are close to people who have tested positive for COVID-19 alarm.

##### Case Study 1: Health Code

**Figure 13:**
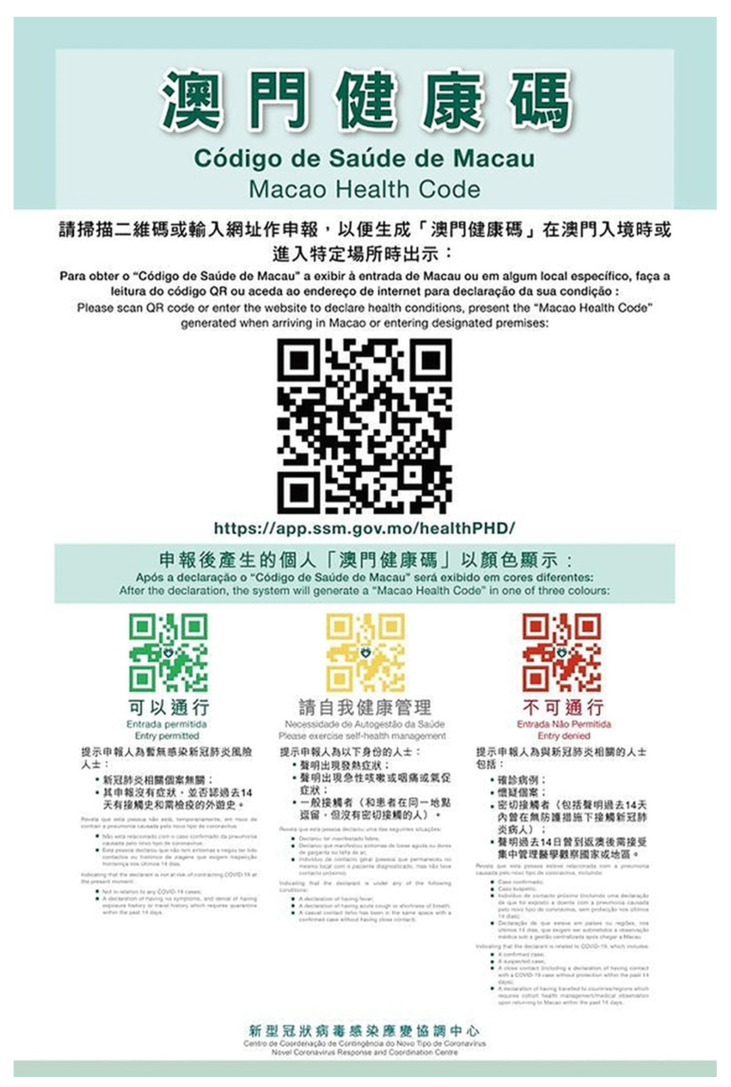
An example of health code in Macao, China [193].

Health Code (HC) is one of the main application scenarios of big data [190,191]. In China, HC is served as a digital health certificate during the epidemic. It can be used in public places (including public transportation, shopping malls, supermarkets, airports, stations, etc.) (Figure 13). Its data comes from a big data platform, which can clearly track users’ personal tracks within 14 days, including traffic data, operator data, and financial institution payment data [192]. The application scenarios of HC cover community management, business resumption, urban transportation, school opening, drug purchase registration, supermarkets and shopping malls. In the prevention and control of the COVID-19 pandemic, using HC realizes efficient management of the flow of people, improves the efficiency of inspections in crowded places such as office buildings, shopping centers, subways and railway stations, and avoids excessive contact and gathering of people.

##### Case Study 2: Infrared Thermal Imaging Thermometer

**Figure 14:**
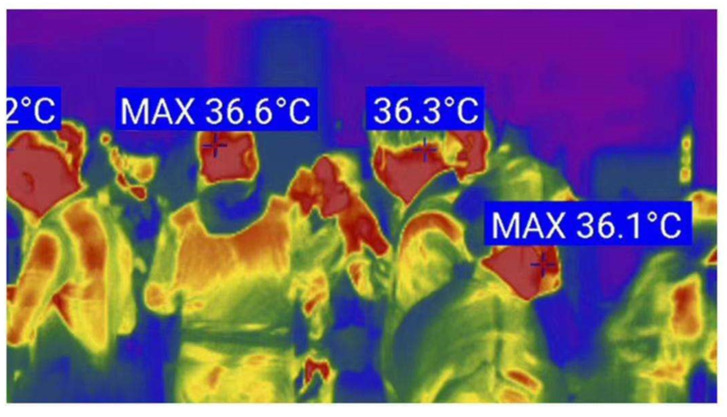
An example of a thermal temperature scan for group temperature measurement. [168].

The infrared body temperature detector meets the non-contact temperature measurement needs during the COVID-19 pandemic and is widely used in crowded public places (Figure 14). This can not only reduce the direct contact between the management personnel and the tested personnel, thereby reducing the possibility of the spread of the epidemic, but also improve the detection speed and the efficiency of personnel passing through high temperature crowds in public places. Compared with traditional temperature measurement methods, infrared geothermal imaging thermometers have three advantages when measuring human body temperature: First, infrared thermal imaging cameras are remote noncontact temperature measurement, without contacting the person to be measured, and preventing personnel cross infection. Second, the infrared thermal imager captures the body temperature automatically. As long as the person under test passes through the field of view covered by the thermal imager lens, the infrared thermal imager can complete body temperature detection without affecting the traffic efficiency of the crowd. Third, thermal imaging cameras measure temperature in a large area. Compared with infrared thermometers and forehead guns that measure temperature one by one, infrared thermal imaging cameras achieve high efficiency temperature measurement, which is especially suitable for crowded places.

### 4.2. Digital Technology in Diagnosis and Treatment

#### 4.2.1. Telemedicine

With the rapid spread of the epidemic, how to improve the diagnosis efficiency of hospitals is a key issue [194]. Many technology companies provide medical institutions with intelligent medical image analysis technology [195]. In Sichuan, based on 5G dual gigabit networks, ZTE and Sichuan Telecom helped West China Hospital of Sichuan University and Chengdu Public Health Clinical Medical Center successfully completed two remote consultations for acute and severe patients with COVID-19 [196]. In the United States, the development achievements of digital medicine have been verified during the epidemic. Virtual care platforms using video conferencing and digital surveillance have been used worldwide to provide patients with telemedicine services to reduce their exposure to SARS-CoV-2 in medical institutions. The digital technology is used to provide remote care for patients with chronic diseases or COVID-19 disease at home. If implemented and delivered properly, virtual care can increase the accessibility of medical services during hospitalization [197].

5G technology has profoundly changed the traditional diagnosis and treatment model. Telemedicine is widely used during the epidemic, including remote consultation, remote consultation, remote training, remote rounds, remote surgery, and remote monitoring. 5G communication technology greatly improved the transmission quality of images and other data in remote consultations, helping to reduce the risk of misdiagnosis. Compared with the traditional diagnosis and treatment modes, using 5G for remote video consultation in hospitals has the following advantages: (1) Through remote consultation, the problem of unbalanced allocation of medical resources in some regions can be alleviated, and effective allocation of medical resources is realized; (2) Using 5G robots for remote consultations, remote rounds, and disinfection services can not only deliver medicines and daily necessities to patients, but also assist nurses in transporting medical equipment and equipment, and processing garbage in wards, which greatly alleviates the shortage of medical staff pressure; (3) Reduce the contact between doctors and patients through the online consultation platform, which not only improves the efficiency of medical treatment, but also reduces the risk of cross-infection of personnel contact.

Traditional remote consultations use wired connections to transmit audio and video information, which is not only expensive in construction and maintenance, but also poor in mobility, making it difficult to meet the needs of consultations in various complex scenarios such as wards and emergency vehicles during the epidemic. Remote consultation needs real-time transmission of patient video and patient images. With the high-speed characteristics of 5G network, it can support 4K/8K remote high-definition consultation and high-speed transmission and sharing of medical image data, so that experts can carry out consultation anytime and anywhere and improve the diagnostic accuracy and guidance efficiency. Since the outbreak, 5G remote consultation has been applied in many hospitals [198,199]. In Wuhan Union Hospital of China, the 5G remote consultation platform can not only realize the consultation between various areas in the hospital, but also realize the interconnection between it and other hospitals. In Huoshenshan Hospital located in Wuhan, medical staff who are fighting on the frontline use 5G to conduct remote consultations with senior medical experts in Beijing through remote video connections. At the same time, telemedicine reduces the risk of infection caused by foreign medical experts having to work in the epidemic area.

Singapore is encouraging the use of telemedicine for chronic diseases such as diabetes, depression and bipolar disorder. In Canada, the number of video visits from clinicians to patients increased from approximately 1,000 visits per day in February 2020 to 14,000 per day in mid-May. The Australian government has launched a home-buying service to allow vulnerable groups in need to order prescription drugs remotely without going to a pharmacy. Philips’ Vital Watch eICU program can remotely monitor the intensive care unit from the monitoring center, thereby helping hospitals solve the current shortage of clinicians in the area [200].

#### 4.2.2. Remote Health Management

The guidelines issued by the WHO point out that suspicious patients with mild symptoms are recommended to undergo home isolation when medical resources are insufficient. The Internet and AI connect patients with medical service providers such as Internet hospitals and offline hospitals to provide patients with convenient, scientific and comprehensive home care knowledge and support services. Combining remote and home care services and adopting a hybrid care model (online + offline) to provide patients with convenient medical care services, provide medical services for areas with low outpatient services, and provide medical support for people with limited mobility.

In China, the People’s Daily health client, the Medical Federation Internet Hospital, and the China Red Cross Foundation jointly released an online medical service platform —“New Coronavirus Pneumonia Out-of-Hospital Family Prevention and Care Service”. The service uses smart hardware to monitor the entire family 7*24 h, and timely monitor the body temperature, blood oxygen saturation, C-reactive protein and other important indicators of the quarantined person. At the same time, the service unites nearly 3000 professional doctors to provide patients with remote consultation and consultation services online, guiding the correct plan for home isolation and protection throughout the process. If necessary, doctors will provide electronic prescriptions online and deliver medicines to your door to reduce the risk of infection when going out. This service can better assist medical institutions and administrative departments to control the epidemic and reduce and reduce the chance of virus cross infection. For non-coronavirus patients suffering from common diseases and chronic diseases, the participation of digital technology can provide continuous online monitoring, diagnosis and treatment services to help patients avoid exposure to the population and reduce the risk of cross-infection.

European countries have made many efforts in this regard [201]. For instance, in Finland, many hospitals established patient-centric digital medical services, called health villages. This online platform enables people to use simple medical equipment to manage personal medical care and send relevant data to professionals. It provides a more simplified method of healthcare, saving patients and doctors’ time to the greatest extent. In Sweden, the authorities have developed a platform for medical staff to report real-time data on the number of COVID-19 patients, personal protective equipment, staffing, ventilator usage and other resource information. This information has been shared with health care institutions across the country to track the status of facilities, allocate health care resources and increase hospital bed capacity [202]. During COVID-19, Germany launched a smart watch application that can collect users’ pulse, body temperature and sleep pattern data to screen for infection. The data from the application can be displayed on an online interactive map for the competent authorities to assess the possibility of COVID-19 on a nationwide scale. These extensive digital medical interventions have kept the per capita mortality rate in Germany at a low level [203].

#### 4.2.3. Ai-Assisted Diagnosis

CT imaging has proven to be an efficient method for screening for COVID-19. As CT is used as the basis for the diagnosis of COVID-19, the number of patients scanned by hospitals has also shown a blowout. It is understood that confirmed patients need 400 CT images, which means that radiologists need to read at least 40,000 CT images a day, which puts tremendous pressure on medical staff. Therefore, AI technology is applied to assist in the analysis of image data to help doctors diagnose the condition [204,205,206]. AI technology can shorten the 5 to 10 min CT reading process to less than 1 min and improve the reading efficiency by nearly 10 times. Compared with manual diagnosis, the biggest advantage of AI-assisted algorithm lies in the speed of image reading, which not only reduces the time for doctors to read images, but also improves the efficiency of clinical diagnosis and treatment [207]. Moreover, the AI system can complete the segmentation of lungs, lung lobes, lung segments, and lesions in seconds, automatically mark the lesions, and calculate the infection ratio of each lung segment, providing doctors with detailed lung infection indicators for patients. The AI-assisted diagnosis system also has the function of grading the severity of the disease and predicting the critical illness.

The European Union has increased its investment in AI technologies, with the aim of using them to accelerate the diagnosis of COVID-19 and monitor the scale of the outbreak in real time [208]. European hospitals apply AI programs to analyze images of patients’ lung infections and assist doctors in making decisions. In the field of radiology, AI tools improved the speed and accuracy of COVID-19 diagnosis by quickly analyzing a large number of medical images. The introduction of AI tools reduced the workload of medical staff during the epidemic and eased the shortage of medical staff. Moreover, the high efficiency of AI can help hospitals treat patients faster while reducing the risk of cross-infection.

#### 4.2.4. Intelligent Robots

Considering the highly contagious nature of COVID-19, minimizing the risk of hospital infection is essential to protect medical staff and win the fight against COVID-19. Intelligent robots suitable for hospital scenarios are widely used to replace some high-risk jobs of medical staff [209,210]. For instance, voice robots based on AI technology are used to provide medical services and collect personal information [211,212]. Voice robots can be used for customer service and simple consultation work, reducing the work pressure of medical staff [213]. On the other hand, voice robots can assist people who do not use smartphones to complete personal information collection. They use voice recognition to convert the collected personal information into text, and then aggregate it into data [214]. In addition, the voice robot is also equipped with an intelligent analysis system to help doctors complete remote diagnosis of patients, reducing the possibility of cross-infection between medical staff and patients [215,216].

In medical institutions in Shanghai and Wuhan, disinfection robots are used to replace manual disinfection. They complete the disinfection by turning on the ultraviolet disinfection lamp on their side and turning on the disinfectant spray on the top of their head. The disinfection robot can realize autonomous navigation and autonomous movement, so as to efficiently and accurately perform disinfection and epidemic prevention without blind spots in the room. Disinfection robots reduces the possibility of infection and transmission of disinfection personnel. Compared with the traditional manual disinfection method, the disinfection robot has a large disinfectant capacity and a long working time. It can work continuously for more than 3h at a time, which greatly relieves the pressure of insufficient medical personnel and improves work efficiency.

The emergence of distribution robots and drones made an important contribution to reduce the risk of infection between medical staff and patients. In the epidemic area, delivery robots are used to deliver food, medicine, water and other daily necessities to people in isolation wards. These smart devices integrate unmanned driving technology and 5G communication network, and can autonomously identify maps, autonomously identify working environments, and plan the best delivery path to complete the point-to-point delivery of materials. Before the robot goes on duty, the staff plan a reasonable route and set the movement mode for the robot according to the specific conditions of the floor and room. With the support of artificial intelligence, the delivery robot can automatically open and close doors, take elevators, avoid obstacles, and charge independently through the intelligent dispatch of the control center. At the same time, it can also realize real-time video monitoring and interaction of various wards. It is understood that one delivery robot is equivalent to the work of three delivery staff, which greatly reduces the safety risks of clinical staff and effectively avoids cross-infection.

In Europe, the European Commission has provided 200 disinfection robots to European hospitals to protect medical personnel from infection [217]. These robots are supported by AI technology and can autonomously complete virus elimination in medical places. They can use ultraviolet rays to disinfect the ward within 15 min to help prevent and reduce the spread of the virus.

##### Case Study 1: WeDoctor

WeDoctor is an internationally leading intelligent digital health platform headquartered in Hangzhou, China. WeDoctor is a medical Internet platform that provides “online + offline” medical services. WeDoctor has established medical partnerships with more than 2700 key hospitals and 240,000 doctors in 30 provinces and cities across the country. Currently, it has more than 160 million registered users. As an Internet hospital, the WeDoctor platform provides services such as free virtual consultation, online self-examination, remote consultation and family medical guidance to the public through remote technology during the COVID-19 pandemic. WeDoctor alleviated the shortage of offline medical resources, met the medical needs of various patients, and made efforts to alleviate the epidemic.

In addition, WeDoctor also provides a “mobile hospital” service to meet patients’ offline treatment needs. This service refers to medical vehicles equipped with intelligent inspection and testing systems. It is equipped with HIS health maintenance system, AI intelligent auxiliary diagnosis system and medical material supply system through cloud services. The doctors in the mobile hospital are all professional medical staff and can independently complete diagnosis and treatment services for 100 diseases. In terms of hardware facilities, these mobile hospitals can provide blood oxygen, blood routine, urine routine, B-mode ultrasound examination, ECG and other medical tests. Mobile hospitals provide basic medical services to community residents, which not only reduces population movement, but also reduces the risk of virus transmission.

##### Case Study 2: Left-Hand Doctor APP

The Left-Hand Doctor Internet Hospital has made important contributions to China’s epidemic prevention and control. The left-hand doctor is a general-medicine robot doctor who can support natural language conversations and provide patients with consultations, medication, and medical knowledge questions. Based on AI technology and big data algorithms, the left-hand doctor platform has created a borderless digital health service network, integrating the fragmented health management service industry, medical industry, and pharmaceutical industry into an Internet platform, which fundamentally improves the efficiency and efficiency of the medical industry. According to reports, the left-hand doctor open platform has served more than 300 industry customers, including pharmacies, mobile medical companies, Internet platforms, and top three hospitals, serving nearly one million people every day.

**Figure 15:**
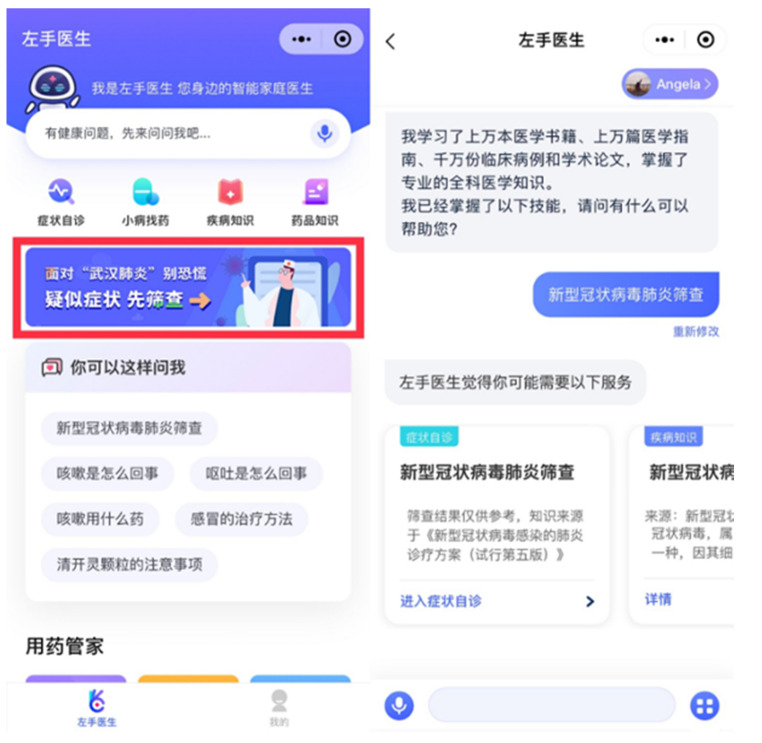
Epidemic consultation page sample.

**Figure 16:**
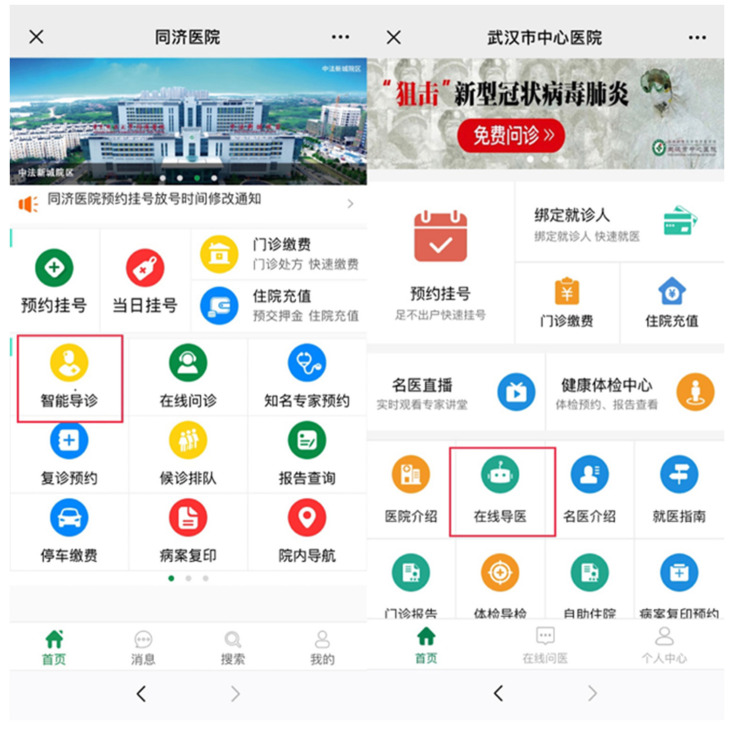
Community monitoring system page [218].

After the COVID-19 outbreak, the left-hand doctor quickly launched the “Outbreak Screening” function (Figure 15) and the “Community Monitoring system” (Figure 16). When users have fever, cough, fatigue and other symptoms suspected of COVID-19, the “epidemic screening” function can simulate the doctor’s consultation process and give conclusions and recommendations for diagnosis and treatment. This function prevents the public from blindly seeking medical treatment during the epidemic and reduces cross-infection to a certain extent. The “community monitoring system” uses artificial intelligence technology and big data methods, relying on WeChat mini-programs to help communities implement health monitoring of personnel and scientifically determine close contacts. Moreover, it also uses the mobile phone to monitor the health status of close contacts on a daily basis to realize intelligent management and improve the efficiency of epidemic management.

### 4.3. Digital Technology in Covid-19 Epidemic Management

#### 4.3.1. Real-Time Epidemic Monitoring System

Epidemic trend monitoring is the prerequisite and basis for epidemic prevention and control [219]. During the COVID-19 pandemic, cloud computing artificial intelligence and big data technology promoted the transition of public health big data from passive observation to active monitoring and early warning [220,221,222]. Experts indicated that advanced information technologies such as artificial intelligence and big data play an important supporting role in public health surveillance [223]. They have information collection, empowerment processing and analysis capabilities, which can assist decision-making departments in early warning and risk assessment of the situation of infectious diseases, thereby improving the accuracy, timeliness and effectiveness of public health surveillance [224]. More concretely, big data analysis combined with artificial intelligence algorithms are used to predict the number of infections, and the overall degree of infection of the epidemic can be assessed through statistics and analysis of the number of confirmed cases. More importantly, trend forecasting can assess the overall trend of the epidemic, confirm whether there will be an inflection point, and when will it occur [225]? These forecast figures are of great significance for lifting the lockdown and resuming production.

**Figure 17:**
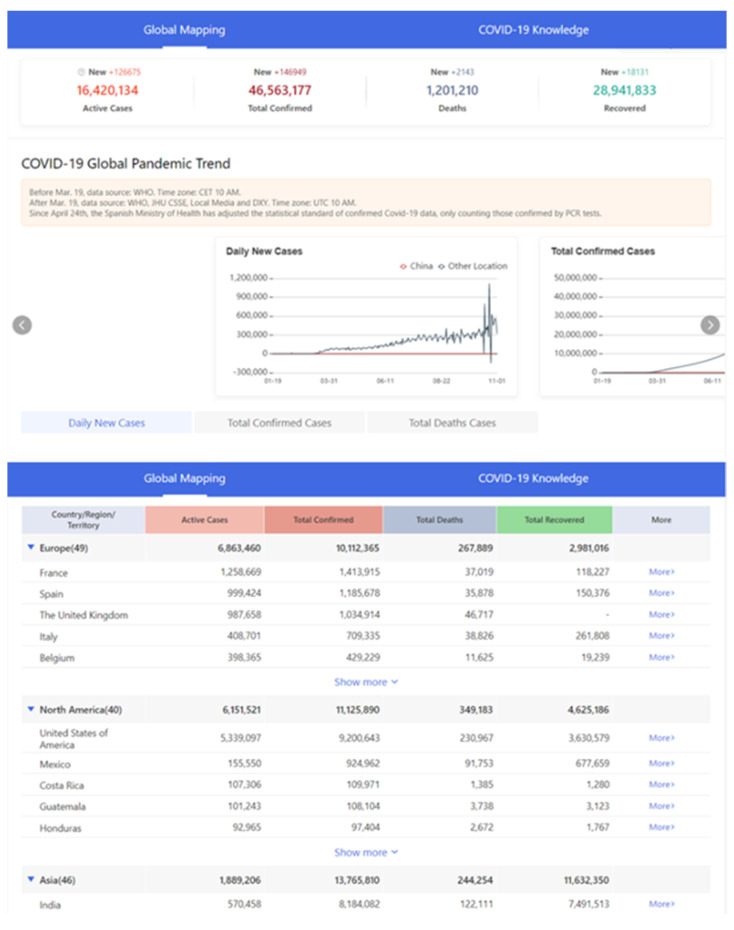
An example of real time report of COVID-19 in China [230].

Trend prediction is an important link in transforming passive prevention into active prevention, and it is also a prerequisite for scientific decision-making [226]. Researchers use mathematical model technology, big data analysis technology and machine learning technology to accurately describe the changing laws of the epidemic, thereby helping the epidemic management department to actively and effectively control the epidemic [227,228]. In China, many provinces made full use of big data, AI, cloud computing and other digital technologies to continuously innovate epidemic surveillance methods [229]. The epidemic monitoring system can dynamically display the latest information such as changes in confirmed cases, the cumulative cases of different types, the prediction of the number of confirmed cases, the comparative analysis of epidemic development trends, and the cure rate (Figure 17). The obtained data provides decision-making basis for implementing classified management and control of the COVID-19 epidemic.

Europe uses satellites to monitor and mitigate the impact of the COVID-19 outbreak. Their satellite navigation system and earth observation system are open to the public, and the observed data can provide decision support for mitigating the epidemic [231]. European countries have combined AI with data from space to draw real-time dynamic maps related to the epidemic. The content of these maps includes real-time traffic conditions in important cities, the location of key medical facilities, etc.

The digital visualization tools not only provide data support for epidemic prevention and control, but also fully protect the public’s right to know at home and abroad, which plays a positive role in enhancing scientific prevention and control knowledge and awareness. Using big data technology, the epidemic monitoring platform constructs a dynamic map of the epidemic situation in the city and region, which helps restore the entire process of the urban epidemic and improve the epidemic prevention and control process. Intelligent analysis and prediction provide intuitive support for government decision-making, emergency management, and resource scheduling, helping decision-makers understand the development and situation of emergency events in the region based on key data and dynamic changes, so that they can quickly make decisions [232]. In addition, timely and accurate information exchange is an important means to ensure public emotional stability and social order during the COVID-19 pandemic, as well as an important way for the government to strengthen epidemic management. News released by digital tools has a wide coverage and strong timeliness. It not only provides real-time epidemic updates, but also comes with official information instructions, which improves the transparency and credibility of information, and reduces misunderstandings and rumors.

#### 4.3.2. Medical Materials Online Allocation System

To curb the spread of the new coronavirus, countries affected by the epidemic adopted strict lockdown measures to block contact between people and prevent crowds from gathering. The reduction of economic production activities and traffic restrictions caused the supply of medical supplies to face great challenges. Since the outbreak of the COVID-19 pandemic, due to the lockdown and the continuous growth of patients, the lack of medical supplies and the shortage of medical staff have become important obstacles to the fight against COVID-19 [233,234]. Improving the supply guarantee capacity in a short period of time become a top priority to combat the epidemic. Intelligent supply chain management through big data and intelligent scheduling of medical protection resources can effectively alleviate the shortage of medical supplies and realize the optimal allocation of medical resources [235,236,237].

Using the Internet platform, the collection, integration, distribution and matching of donation information can be completed online, which greatly improves the efficiency of resource matching. For example, China’s Ministry of industry and information technology established a national key medical material support and scheduling platform to collect, count, analyze, monitor and dispatch the production capacity, output, inventory and transportation of various materials. Some companies use Internet big data to develop medical supplies platform. Haier launched COVID-19 medical materials information sharing resource platform on the industrial Internet platform, which can timely and accurately update the supply and demand information of epidemic prevention and control materials, help production enterprises to effectively match the demand of epidemic areas, and provide data support for the government’s material allocation [238]. The “National Big Data Public Service Platform for Epidemic Prevention and Control Materials Industry” launched by JD has established a comprehensive service system for demand collection, overall scheduling, and logistics tracking for prevention and control materials [239]. These material guarantee platforms can not only realize the registration, inventory and management of materials, and find out the overall supply of prevention and control materials; but also realize the precise collection and supply-demand matching of prevention and control material demand of various departments and solve the material shortage dilemma.

Emerging blockchain technology also made important contributions to fight against the pandemic. The US IBM’s fast supplier connection network uses distributed accounting technology to help manufacturers of relief supplies establish contact with demanders. IBM’s supercomputer “Summit” has simulated the compounds of more than 8000 drugs for researchers within a few days, and it would take months to use traditional computers.

##### Case Study 1: COVID-19 Map of Johns Hopkins University

**Figure 18:**
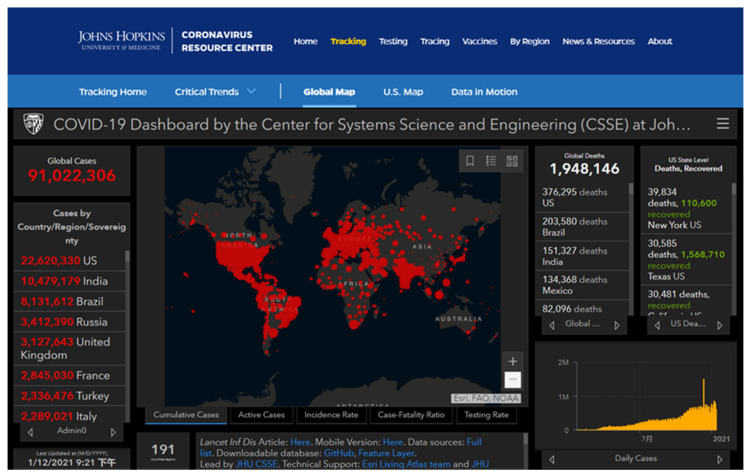
Global map of COVID-19 cases on January 12, 2021 [240].

Johns Hopkins University in the United States developed a coronavirus dashboard and a COVID-19 map, which can provide the latest images of COVID-19 cases and deaths from around the world (Figure 18). After the COVID-19 outbreak, the Coronavirus Resource Center of Johns Hopkins University began tracking the global cases of the COVID-19 epidemic from hundreds of data sources around the world and updated the latest changes daily. This big data platform has become one of the most cited databases related to the epidemic.

##### Case Study 2: Baidu Map

**Figure 19:**
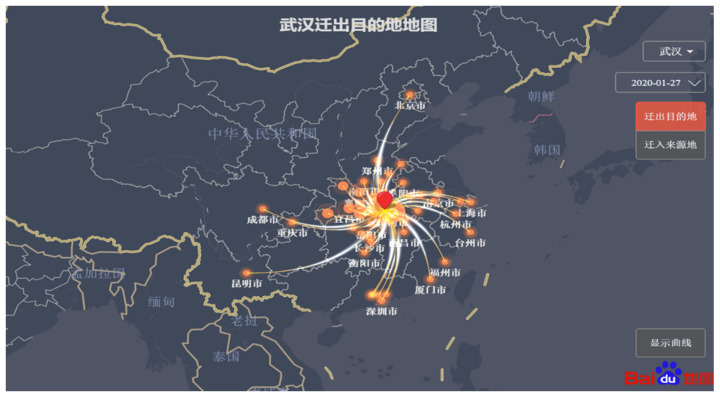
A screenshot of real-time Wuhan’ residents emigration destination of Baidu map [245].

Baidu Map, Tencent and China Mobile successively released real-time population migration data based on AI, providing data references for epidemic management in different regions [241,242]. The outbreak of COVID-19 coincides with China’s Lunar New Year holiday, a period of mass migration. The “Baidu Migration Map” big data platform launched by Baidu map uses massive location service data to display the trajectory and characteristics of population migration around the Spring Festival in a complete, dynamic, instant, and intuitive manner [243,244]. During the Spring Festival, users can intuitively understand the short-term population flow between cities. The Baidu Migration Map used real-time, comprehensive, and authoritative data information to ensure that users can travel accurately, efficiently and safely, and inject science and technology into strengthening epidemic prevention and control in various places and restoring social production and life order (Figure 19).

At present, the platform has opened more than 300 urban population migration sources, destinations, migration scale index, migration scale trend, urban travel intensity and other data indicators. The platform uses spatiotemporal big data, artificial intelligence technology, and geographic information processing technology to intelligently and visualize complex urban population migration data, so that users can understand the flow of people in the outbreak area in time.

### 4.4. Digital Technology in Drug Development

#### 4.4.1. SARS-CoV-2 Gene Sequencing

A comprehensive understanding of the characteristics of the virus is essential to prevent infection and control the scale of the outbreak. Covid-19 is caused by the SARS-CoV-2 virus, which is a new type of virus. Therefore, it is necessary to conduct in-depth research on the SARS-CoV-2 virus, including the mutation prediction and transmission mode of the virus. Carrying out epidemiological research on SARS-CoV-2 is of great significance for better control of the epidemic and treatment of patients.

The gene sequencing of SARS-CoV-2 and the study of its protein structure are important contents of epidemiological research. Researchers use advanced digital technologies such as artificial intelligence and supercomputers to quickly calculate and analyze related stored data. Digital tools play an important role in epidemiological research. Using supercomputers to compare sequencing results with other viruses significantly reduces the time required for genetic sequencing of SARS-CoV-2. The intelligent calculation model can directly display the protein structure of SARS-CoV-2, which helps researchers understand its pathogenesis and transmission mechanism. More importantly, big data calculations can predict the mutations of viral proteins and help us grasp the possible mutation trends of viruses in the future.

Many technology platform companies opened up AI computing power to medical and epidemic prevention scientific research institutions for free for the first time, saving time for virus gene sequencing. For example, on January 30, Baidu announced that it would open the linear time algorithm LinearFold and the world’s fastest RNA structure prediction website for free to genetic testing institutions and scientific research centers around the world, with the purpose of helping them improve the prediction speed of the COVID-19 RNA spatial structure. Baidu provides AI technology support and billions of computing resources to help disease control institutions and other research institutions accelerate the development of effective drugs against COVID-19. Besides, MGI Tech in Shenzhen, China has developed the first batch of high-throughput sequencers to help researchers around the world perform gene sequencing and assembly of SARS-CoV-2 at a faster speed. European countries have established a big data platform related to covid-19 to provide data support for the epidemic management plan. The COVID-19 data platform was launched in April 20th by European Commission to provide researchers with an open digital platform for Europe and the world to collect, store and share new coronavirus gene sequences, protein structure and other research data [246].

#### 4.4.2. Drug and Vaccine Development

Currently, researchers are using different technology platforms to develop COVID-19 vaccines. Note that the development of new drugs and vaccines requires a large amount of data analysis, large-scale literature screening and scientific supercomputing work. AI and cloud computing have been effectively used in drug research and development and made important contributions to epidemic prevention and control [247,248]. Researchers use AI and big data database to create models to predict the development and spread of the COVID-19, help medical staff improve prevention and control measures, and slow down the further spread of the epidemic [249,250]. AI plays an irreplaceable role in improving the efficiency of virus detection and speeding up drug development [251,252]. With the help of AI algorithms, an automated whole-gene detection and analysis platform can shorten the genetic analysis of suspected cases to half an hour and can accurately detect virus mutations. AI technology greatly improved the speed and accuracy of the diagnosis of suspected cases and reduced the risk of suspected cases being infected again. AI has also been invested in pharmacology and toxicology research, protein screening, and new drug development, which has greatly accelerated the development of vaccines and other drugs.

Cloud services can provide AI computing power, support viral gene sequencing, new drug development, protein screening, etc., and help scientific research institutions shorten the development cycle and reduce costs. The combination of big data, AI and computing power is critical to the development of SARS-CoV-2 drugs and vaccines. These advanced digital technologies have accelerated the vaccine development process and provided technical support for data calculations in epidemiological research. For example, in China, the Global Health Drug Research and Development Center (GHDDI), together with the School of Pharmacy of Tsinghua University, uses the GHDDI AI drug research and development and big data platform to conduct data mining and integration for the historical drug research and development of SARS, MERS and other coronaviruses, focusing on multiple potential targets and Mechanisms to carry out drug screening simultaneously [253]. The Global Health Drug R&D Center has also opened up technical platforms and drug R&D resources such as artificial intelligence drug R&D and big data sharing platform to scientific researchers. Internet companies such as Tencent cooperated with the National Supercomputing Center in Shenzhen to provide a platform for the development of SARS-CoV-2 drugs and vaccines. This platform has supercomputing capabilities to support large-scale epidemiological research and drug screening.

Italy’s CINECA National Supercomputing Center is using supercomputers to perform simulation calculations on the new coronavirus, screening 1000 or even fewer compounds from millions of compounds that are most likely to curb the spread of the virus, so as to test which drug is most effective in the laboratory. In June 2020, Exscalate4CoV, a consortium from Italy, announced that the registered generic drug for the treatment of osteoporosis may be an effective method for the treatment of coronavirus [254]. This is the result of their research with the help of the supercomputing platform invested by the European Union. On October 27, the Italian drug administration AIFA approved a clinical trial of raloxifene in patients with mild symptoms caused by coronavirus.

## 5. Challenges

### 5.1. Data Lag

Epidemic trend prediction is one of the most important application scenarios of big data during the COVID-19. Generally, big data analysis is to establish a predictive model through a large number of collected data samples, so as to perform model fitting and predictive analysis. However, when the amount of data is insufficient or inaccurate, the results of predictive analysis fitted by existing data are not accurate [255]. In the early stage of the pandemic, big data analysis did not have high credibility for the results of pandemic prediction, mainly due to the following reasons: (1) It is difficult to determine relevant model parameters; (2) There is insufficient data for cross-validation and optimization; (3) The complexity of COVID-19 causes the existing models to fail to accurately feedback the characteristics of the virus transmission.

The data lag is an important reason why big data models cannot effectively evaluate COVID-19. After the outbreak of the pandemic, the data of big data platform mainly came from users who actively reported personal information. Therefore, there is a lag in pandemic data, leading to a lack of big data support for many decisions, thus missing the opportunity to take non-pharmaceutical intervention measures earlier. The current system is a linear system based on process processing, rather than a multivariate system based on big data. Linear systems can only make decisions and speak out through layer-by-layer reporting and transmission and cannot understand the real information in time. Establishing an intelligence big data system for public health is critical to responding to health emergencies.

During the COVID-19 pandemic, it is very difficult for big data platforms to collect and process trajectory data. On the one hand, there are challenges in obtaining individual trajectory data [256]. At the beginning of the outbreak, individual trajectory data was mainly obtained through a wide range of cameras. However, the low camera coverage, the structured analysis of video data, and the imprecision of distance and time information make the data obtained more suitable for trajectory tracking of a single person, rather than crowd tracking. In comparison, the location data provided by smartphone operators is more convenient and accurate. However, it is inevitable that the quality of location data provided by operators is uneven, and problems such as missing original data, inaccurate data, and incomplete data storage will affect the use of trajectory data.

On the other hand, how to effectively analyze trajectory data is another application difficulty. Trajectory big data is used for trajectory collision detection. The process of trajectory collision detection is complex, not only involving a large number of algorithms, but also including physical model calculation and machine learning model inference. So far, teams that can provide theory, engineering and service capabilities, and has experience in location trajectory big data analysis is very rare in China. Moreover, the current technical level is difficult to define the scene boundary of the big data analysis of the pandemic trajectory. If no constraint conditions are defined when building a model using trajectory big data, the solution space will be very large and accurate results cannot be obtained. This is the reason why trajectory collision analysis through trajectory big data in the early stage of the outbreak did not achieve good results [257,258].

### 5.2. Data Fragmentation

Data fragmentation is a difficult problem facing the field of digital healthcare. Medical data volume is huge, with rich types the diversity of data types leads to the diversity of data structure, which increases the difficulty of data collection. On the other hand, medical health data is throughout a person’s life, and most of them are related to time. Since electronic medical records have not yet been fully popularized, and some data are derived from manual records, it will cause deviation and incompleteness of the recorded content. Since the collection and processing of medical and health data are often not closely connected, the data in the medical database is large in scale but still difficult to be fully integrated, and the degree of data fragmentation is serious [259].

### 5.3. Personal Privacy

In the process of fighting against covid-19, digital technology uses a lot of personal data. Whether disclosing personal information related to the epidemic will threaten the privacy and safety of the public has become the focus of attention [260]. During the COVID-19, using big data technology for virus traceability and personal trajectory tracking is an effective way to prevent the spread of the pandemic. There have been many acts of collecting personal information in the early investigation of the pandemic, subsequent isolation and prevention, and the deployment of material requirements, including filling in offline paper forms and collecting data online through applications. These data collection methods may involve the issue of personal data privacy, making the public very worried about personal privacy data being leaked and illegally collected and used. On the one hand, the more data types and data analyzed, the easier the aggregation of various information may be related to specific personal information [261]. On the other hand, the complicated network environment and the real-name authentication system make the associated individuals who use the device will be continuously matched. Some big data companies developed various digital tools based on big data technology, such as health code applications and real-time update systems for pandemic data. These applications can automatically obtain the user’s geographic location, movement trajectory, personal information, etc., causing people to worry about personal privacy leakage.

With the rapid development of digital health, technologies such as electronic medical records, mobile medical care, and AI medical imaging continue to spread. In this process, medical data showed the characteristics of explosive growth, and the enormous abundance of data also brought people’ concerns about privacy. At the same time, there are more and more medical data breaches. Many institutions and technology companies will use consumer data for commercial processing. Some organizations may leak consumer data without the consumer’s consent, triggering consumers Concerns about the privacy and security of his personal medical data. Consumers worry that their health may be unreasonably used, which will have an impact on their lives. Therefore, consumers are increasingly protecting their privacy. In this environment, consumers will no longer be willing to over-share their data. This will cause great inconvenience to medical data demanders such as scientific research personnel and healthcare institutions.

Data security and privacy issues may arise in every link of medical data collection, storage, application, and destruction. For example, during the collection process, some institutions collect data privately without the patient’s consent, which brings great data hidden dangers; during the storage process, weak data protection measures can lead to malicious theft of data. At the same time these problems appear, scholars are also actively looking for corresponding solutions. For example, data privacy security protection technologies such as key encryption, access control, and anonymity technologies for patient data have been proposed [262,263].

### 5.4. Digital Security Breaches

Digital security breaches are one of the important obstacles to the rapid development of digital healthcare. Digital security vulnerabilities mainly come from medical equipment problems. Although medical equipment has improved the quality and feasibility of medical care, the digital loopholes it creates can have a negative impact on individuals’ lives.

The current medical equipment is connected through the network and is no longer an independent system. While this improves convenience, it also increases the possibility of attackers stealing data. Attackers can conduct network attacks through multiple communication media (including software and hardware vulnerabilities, Ethernet communication protocols, personal computer or smartphone applications, and applications that connect to the gateway via Wi-Fi), causing digital security vulnerabilities [264]. When an attacker tries to modify the hardware of the control device, if the security mechanisms such as identity verification and access control are insufficient, the attacker can unfairly access sensitive health-related information and intercept user personal information without permission, causing privacy and security risks. Medical institutions should pay attention to and avoid digital security loopholes. The rapid development of digital healthcare has led to the high value of huge-scale medical data being noticed by more and more people. If the data collection equipment is attacked, the data will be stolen.

### 5.5. Regulatory System

At present, there is no clear legal and regulatory framework in the field of digital health. Most medical technologies are not regulated by medical institutions. However, studies have shown that the lack of “approval” from official governments or authoritative organizations will hinder market adoption [265]. By 2017, 120 countries in the world have special personal information protection laws [266], but many countries lack special laws and regulatory rules on medical and health information. Failure to achieve data protection of medical data and medical equipment will directly result in the insecurity of the privacy, integrity and availability of consumer data. Therefore, each country should establish a systematic, scientific, and effective regulatory system to protect consumers’ autonomy in the medical field.

## 6. Outlook and Implications

The new generation of information technologies have made important contributions to the fight against the COVID-19 pandemic [267,268]. The outbreak of COVID-19 has accelerated the development of information technology, especially the innovation of digital technologies such as AI, 5G, cloud technology, and big data [269]. The COVID-19 pandemic boosted China’s informatization construction, accelerated the application of information technology to the medical and health care field, and further broadened the application scope of digital Technologies in the medical and health care field.

In China, in response to the issue of personal privacy protection, the Office of the Central Cyber Security and Informatization Commission issued the “Notice on Protecting Personal Information and Using Big Data to Support Joint Prevention and Control Work”. The “Notice” puts forward clear requirements for protecting personal privacy: (1) Do personal information security protection; (2) Give full play to the role of big data in the pandemic. The document emphasizes that without the consent of the collected person, no unit or individual may disclose personal information such as name, age, ID number, telephone number, home address, etc., except for information that has become insensitive due to the need for joint prevention and treatment. Organizations that collect or possess personal information shall be responsible for the safety and protection of personal information and adopt strict management and technical protection measures to prevent it from being stolen and leaked.

Ensuring data security is one of the necessary obligations of data controllers and processors, especially big data information companies that have mastered special and important categories of data. The big data platform not only needs to establish and improve the internal data protection and data leakage emergency response mechanism, but also have sufficient technical security measures to prevent hacker attacks and avoid the leakage of user privacy information. Take data storage as an example. When a big data company collects a large number of different sample information, it should immediately perform de-identification processing, and separate the de-identified data and the information that can be used to restore the identification of individuals and ensure that the Individuals are not re-identified during the processing of personal information.

## Figures and Tables

**Table 1:** Search strategy for publications on digital healthcare from Scopus.

Topic	Search Strategy	Publications
Digital healthcare	TITLE-ABS-KEY (digital AND healthcare)	8444
COVID-19&Digital healthcare	(TITLE-ABS-KEY (covid-19) OR TITLE-ABS-KEY (2019-ncov) OR TITLE-ABS-KEY (2019 novel AND coronavirus *) OR TITLE-ABS-KEY (2019 novel-cov) AND TITLE-ABS-KEY (digital AND healthcare))	133

Note: * indicates single or multiple characters.

**Table 2:** Top 10 funding institutions by number of publications.

Funding Agency	Country/Region	Type
National Institutes of Health	United States	public funding
National Natural Science Foundation of China	China	public funding
National Science Foundation	United States	public funding
National Institute for Health Research	United States	public funding
European Commission	Europe	public funding
Horizon 2020 Framework Programme	Europe	public funding
European Regional Development Fund	Europe	public funding
Medical Research Council	United Kingdom	public funding
National Cancer Institute	China	public funding
Engineering and Physical Sciences Research Council	United Kingdom	public funding

**Table 3:** Top ten source publications by number of documents.

Source Publication	Country	Subject Area and Category	H-Index	SJR 2019	SNIP 2019
Studies in Health Technology and Informatics	Netherlands	Engineering; Health Professions; Medicine	56	0.267	0.457
Lecture Notes in Computer Science	Germany	Computer Science; Mathematics	356	0.427	0.776
Advances in Intelligent Systems and Computing	Germany	Computer Science; Engineering	34	0.184	0.429
ACM International Conference Proceeding Series	United States	Computer Science	109	0.2	0.333
IEEE Access	United States	Computer Science; Engineering; Materials Science	86	0.775	1.734
BMJ Open	United Kingdom	Medicine	84	1.247	1.359
Communications in Computer and Information Science	Germany	Computer Science; Mathematics	45	0.188	0.403
International Journal of Medical Informatics	Ireland	Medicine	99	0.954	1.958
Journal of Digital Imaging	United States	Computer Science; Health Professions; Medicine	51	0.967	1.641
Proceedings of SPIE The International Society for Optical Engineering	United States	Computer Science; Engineering; Materials Science; Mathematics; Physics and Astronomy	162	0.215	320

**Table 4:** The top nine medical service stocks in the U.S. for gains in 2020.

Securities Code	Market Value (2020/5, Billion USD)	Quote Change (January–May)	Business Profile
LVGO.O	57.74	135.55	Chronic disease management
TDOC.N	128.21	105.69	Telemedicine
SMED.O	1.14	65.88	Medical waste treatment
AHCO.O	12.22	50.27	Respiratory equipment rental and sales
INFU.A	2.3	34.47	Medical Supplies
CATS.O	3.61	30.35	Psychological intervention-Insurance
PRSC.O	8.65	16.52	Pharmaceutical logistics
LHCG.O	50.6	16.27	Hospice care + Home care
AMED.O	62.56	15.74	Hospice care + Home rehabilitation

## Data Availability

Not applicable.
